# Christmas, national holidays, sport events, and time factors as triggers of acute myocardial infarction: SWEDEHEART observational study 1998-2013

**DOI:** 10.1136/bmj.k4811

**Published:** 2018-12-12

**Authors:** Moman A Mohammad, Sofia Karlsson, Jonathan Haddad, Björn Cederberg, Tomas Jernberg, Bertil Lindahl, Ole Fröbert, Sasha Koul, David Erlinge

**Affiliations:** 1 Department of Cardiology, Clinical Sciences, Lund University, Skane University Hospital, Lund, Sweden; 2 Department of clinical sciences, Danderyd’s University Hospital, Karolinska Institutet, Stockholm, Sweden; 3 Department of Medical Sciences and Uppsala Clinical Research Center, Uppsala University, Uppsala, Sweden; 4 Örebro University, Faculty of Health, Department of Cardiology, Örebro, Sweden

## Abstract

**Objectives:**

To study circadian rhythm aspects, national holidays, and major sports events as triggers of myocardial infarction.

**Design:**

Retrospective observational study using the nationwide coronary care unit registry, SWEDEHEART.

**Setting:**

Sweden.

**Participants:**

283 014 cases of myocardial infarction reported to SWEDEHEART between 1998 and 2013. Symptom onset date was documented for all cases, and time to the nearest minute for 88%.

**Interventions:**

Myocardial infarctions with symptom onset on Christmas/New Year, Easter, and Midsummer holiday were identified. Similarly, myocardial infarctions that occurred during a FIFA World Cup, UEFA European Championship, and winter and summer Olympic Games were identified. The two weeks before and after a holiday were set as a control period, and for sports events the control period was set to the same time one year before and after the tournament. Circadian and circaseptan analyses were performed with Sunday and 24:00 as the reference day and hour with which all other days and hours were compared. Incidence rate ratios were calculated using a count regression model.

**Main outcome measures:**

Daily count of myocardial infarction.

**Results:**

Christmas and Midsummer holidays were associated with a higher risk of myocardial infarction (incidence rate ratio 1.15, 95% confidence interval 1.12 to 1.19, P<0.001, and 1.12, 1.07 to 1.18, P<0.001, respectively). The highest associated risk was observed for Christmas Eve (1.37, 1.29 to 1.46, P<0.001). No increased risk was observed during Easter holiday or sports events. A circaseptan and circadian variation in the risk of myocardial infarction was observed, with higher risk during early mornings and on Mondays. Results were more pronounced in patients aged over 75 and those with diabetes and a history of coronary artery disease.

**Conclusions:**

In this nationwide real world study covering 16 years of hospital admissions for myocardial infarction with symptom onset documented to the nearest minute, Christmas, and Midsummer holidays were associated with higher risk of myocardial infarction, particularly in older and sicker patients, suggesting a role of external triggers in vulnerable individuals.

## Introduction

Ischaemic heart disease is declining in high income countries but remains the commonest cause of morbidity and mortality worldwide.^1^ Current understanding is that the disease is multifactorial with predisposing modifiable and non-modifiable risk factors.^1^ ^2^ ^3^ However, studies have also shown that external factors may be involved in triggering the onset of myocardial infarction by eliciting the rupture of unstable plaques. Moreover, Muller et al. presented the circadian variation in myocardial infarction incidence, peaking at 9 am, and Goldberg et al. and Rocco et al. further expanded this investigation by adjusting for time of waking, showing that the risk of myocardial infarction increases during the first 1-4 hours after waking.^4^ ^5^ ^6^ ^7^

Short term risk factors include, but are not limited to, emotional stress, heavy physical activity, cold weather exposure, and air pollution.^3^ ^8^ ^9^ ^10^ ^11^ ^12^ ^13^ ^14^ ^15^ External factors such as earthquakes, hurricanes, and wars, as well as sports events and stock market volatility, have repeatedly been associated with a higher risk of myocardial infarction.^16^ ^17^ ^18^ ^19^ ^20^ Furthermore, studies have shown a peak in cardiac mortality in the Western world on Christmas Day and New Year’s holiday, and during Islamic holidays in countries where these are widely celebrated.^21^ ^22^ ^23^ ^24^ Sir Winston Churchill is thought to have experienced myocardial infarction while visiting the White House during Christmas 1941.^25^ However, most studies have used surrogate variables, such as mortality due to myocardial infarction, ambulance records, death certificates, and administrative data including International Classification of Diseases codes as indicators of myocardial infarction, and as such may introduce bias through misclassification and uncertainties in time of symptom onset. Thus, there is a lack of more granular data with exact time of symptom onset, as well as severity and type of myocardial infarction in a nationwide setting.

We hypothesised that short term risk factors may be associated with a higher risk of myocardial infarction. Furthermore, we hypothesised that the potential risk may differ for electrocardiogram (ECG) subtypes of myocardial infarction: ST elevation myocardial infarction (STEMI) and non-ST elevation MI (NSTEMI). Our objective was therefore to study national holidays, major sports events, and circadian rhythm aspects as triggers of ECG and biomarker positive myocardial infarctions with high quality data on symptom onset documented to the nearest minute, in a large nationwide setting.

## Methods

### Study population

We used the prospective nationwide Swedish Web System for Enhancement and Development of Evidence-Based Care in Heart Disease Evaluated According to Recommended Therapies (SWEDEHEART) registry to identify cases of myocardial infarction in Sweden between 1998 and 2013. The SWEDEHEART registry has been described in detail elsewhere. Briefly, all patients with symptoms of an acute coronary syndrome admitted to a coronary care unit or other specialised facility in Sweden are enrolled.^26^ The registry prospectively collects information on background characteristics, including age, body mass index, smoking status, electrocardiographic findings, and other examinations, interventions, complications, laboratory measures, discharge medications, and diagnoses. The diagnosis of myocardial infarction, including subtypes, is made based on the treating physician’s assessment of the patient at discharge. The date and time of symptom onset are documented to the nearest minute, and we used these as the main variables in this study. All cases of myocardial infarction reported to the SWEDEHEART registry during the study period were included in this study.

### Study design

We identified all cases of myocardial infarction with symptom onset on Christmas/New Year, Easter, and Midsummer holiday. We defined Christmas/New Year holidays as Christmas Eve, Christmas Day, Boxing Day (26 December), New Year’s Eve, New Year’s Day, and the Epiphany. We defined Easter holiday as Good Friday, Easter Eve, Easter Day, and Easter Monday. Midsummer Eve and Midsummer Day (also known as St John’s Day) constituted Midsummer holiday (box 1). Similarly, we identified all myocardial infarctions that occurred during all Fédération Internationale de Football Association (FIFA) World Cup tournaments, Union of European Football Association (UEFA) European Championship tournaments, and winter and summer Olympic Games in the study period. The two weeks before and after a holiday were set as the control period. For sports events, we set the control period to the same period one year before and after the tournament. We performed circadian and circaseptan analyses, with Sunday and 12 am as the reference day and hour with which to compare all other days and hours. The primary outcome was daily count of myocardial infarction, with STEMI and NSTEMI studied independently as secondary outcome measures. We calculated incidence rate ratios comparing incidence rates of a period/time of interest with a control period according to the statistical analysis section. We assessed pre-specified subgroup analyses for all periods of interest. These subgroups were based on sex, age ≥75 versus age <75, smoking status, diabetes, hypertension, coronary artery disease, and patients taking drugs such as β blockers, calcium inhibitors, aspirin, angiotensin converting enzyme inhibitors/angiotensin receptor blockers (ACE-I/ARB), and statins. We conducted a sensitivity analysis of the risk of myocardial infarction on days where the Swedish football team was involved in the FIFA World Cup and UEFA European Championship tournaments. In addition, we conducted sensitivity analyses adjusted for year as a categorical variable to control for time trends in myocardial infarction during the study period.

Box 1 **Holidays in Sweden**

**Christmas**

Christmas is celebrated with the immediate family, and Christmas Eve on 24 December is the main day of Christmas festivities. Celebrations continue on Christmas Day and Boxing Day (25 and 26 December, respectively).

**New Year**

New Year’s Eve (31 December) is generally celebrated with friends. The festivities involve consuming food and alcoholic drink to excess and awaiting fireworks at midnight to share New Year’s resolutions and wish one another a happy new year.

**Easter**

Easter celebration is the first long weekend after winter. Families and friends meet and eat. Eggs are a theme, and children dress as Easter witches.

**Midsummer**

Excepting Christmas, Midsummer is the most important holiday in Sweden. On Midsummer Eve, which precedes the summer solstice, Swedes gather to celebrate by dancing around a maypole, singing, eating, and drinking, often to excess. It is associated with the Christian holiday, the Feast Day of St John the Baptist, on 24 June.

### Statistical analyses

Continuous variables are displayed as mean and standard deviation. Categorical variables are displayed as counts and percentages. We used a χ^2^ test to assess statistical significance between categorical variables. We calculated incidence rate ratios for major holidays, sports events, day of the week, and hour of symptom onset using a Poisson regression model. Calendar week was plotted with a line graph to assess seasonality. We conducted all analyses on complete case data, and only two variables contained missing data exceeding 1%. These were time of symptom onset and smoking status, with 12% and 9% missing data, respectively. We applied family wise error rate within strata (total myocardial infarction, NSTEMI, and STEMI) using the Hochberg method to control for type I errors owing to multiple testing. All statistical analyses were performed using STATA version 14.1 for Macintosh (StataCorp, TX). A two sided P value <0.05 was considered statistically significant.

## Results

### Patient characteristics

During the study period, 283 014 admissions for myocardial infarction occurred and all were included in the study. Baseline demographics are presented in table 1. Overall, 95 176 patients had STEMI on admission. Patients with STEMI were on average four years younger, more often men and current smokers compared with patients with NSTEMI.

**Table 1:** Baseline demographics for study population. Values are numbers (percentages) unless stated otherwise

Characteristics	Total MI (n=283 014)	STEMI (n=95 176)	NSTEMI (n=187 838)
Mean (SD) age (years)	71.7 (12.2)	69.1 (12.5)	73.0 (11.8)
Men	180 205 (64)	63 119 (66)	117 086 (62)
Women	102 809 (36)	32 057 (34)	70 752 (38)
Mean (SD) BMI	26.7 (5.9)	26.6 (7.1)	26.8 (5.2)
Mean (SD) eGFR	68.2 (24.1)	73.4 (23.1)	65.8 (24.2)
Current smoker	55 673 (20)	24 531 (26)	31 142 (17)
**Medical history**			
Diabetes	61 955 (22)	15 806 (17)	46 149 (25)
Hypertension	123 436 (44)	36 306 (38)	87 130 (46)
Coronary artery disease	100 638 (36)	21 280 (22)	79 358 (42)
Myocardial infarction	91 283 (32)	19 345 (20)	71 938 (38)
PCI	32 846 (12)	7574 (8)	25 272 (13)
CABG	23 971 (8)	3711 (4)	20 260 (11)
Chronic heart failure	3373 (1)	573 (1)	2800 (1)
Stroke	17 116 (6)	3935 (4)	13 181 (7)
Drugs at admittance			
β -blockers	120 904 (43)	29 851 (31)	91 053 (48)
Calcium antagonist	51 107 (18)	13 603 (14)	37 504 (20)
Aspirin	123 647 (44)	29 258 (31)	94 389 (50)
ACE-I/ARB	88 592 (31)	20 955 (22)	67 637 (36)
Statins	75 381 (27)	16 978 (18)	58 403 (31)

Diuretics

87 367 (31)

19 180 (20)

68 187 (36)

MI=myocardial infarction; STEMI=ST elevation myocardial infarction; NSTEMI=non-ST elevation myocardial infarction; BMI=body mass index; eGFR=estimated glomerular filtration rate; PCI=percutaneous coronary intervention; CABG=coronary artery bypass graft; ACE-I/ARB=angiotensin converting enzyme inhibitor/angiotensin receptor blocker

### Major holidays

**Fig 1:**
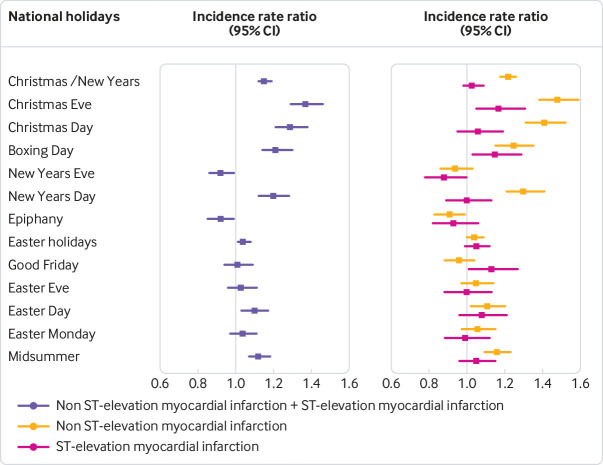
Associated risks of national holidays and myocardial infarction. ST elevation myocardial infarction and non-ST elevation myocardial infarction expressed as incidence rate ratios for all major national holidays. P values after adjustment for multiple testing are presented in table 2

**Fig 2:**
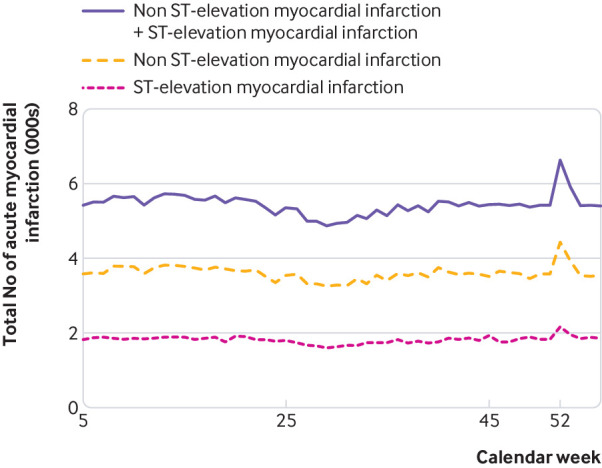
Total number of myocardial infarctions by calendar week, peaking on calendar week 52

A higher risk of myocardial infarction was observed for Christmas Eve, Christmas Day, Boxing Day, and New Year’s Day, but not for New Year’s Eve and Epiphany (table 2, fig 1, fig 2). During the Christmas/New Year holiday the overall risk of myocardial infarction increased by 15% (incidence rate ratio 1.15, 95% confidence interval 1.12 to 1.19, P<0.001). Risk of myocardial infarction on Christmas Eve was higher than on the other days of the holiday (1.37, 1.29 to 1.46, P<0.001) and the risk was higher still after ECG stratification of myocardial infarction, with higher risk of NSTEMI (1.48, l 1.38 to 1.59, P<0.001). Easter holiday was not associated with a higher incidence rate ratio of myocardial infarction, while Midsummer holiday was associated with a higher risk (1.12, 1.07 to 1.18, P=0.001) largely driven by a higher risk of NSTEMI (1.16, 1.09 to 1.23, P<0.001).

**Table 2:** Results, after adjustment for multiple testing

Holidays and sports events	Total myocardial infarction				Non-ST elevation myocardial infarction				ST elevation myocardial infarction		
	Period of interest	Control period			Period of interest	Control period			Period of Interest	Control period	
	Incidence/day	Incidence/day	IRR (95% CI)		Incidence/day	Incidence/day	IRR (95% CI)		Incidence/day	Incidence/day	IRR (95% CI)
**Christmas/New Year**	57.9	50.3	1.15 (1.12 to 1.19)***		40.1	33	1.22 (1.17 to 1.26)***		17.8	17.3	1.03 (0.98 to 1.09)
Christmas Eve	69.1	50.3	1.37 (1.29 to 1.46)***		48.9	33	1.48 (1.38 to 1.59)***		20.2	17.3	1.17 (1.05 to 1.31)
Christmas Day	64.9	50.3	1.29 (1.21 to 1.38)***		46.6	33	1.41 (1.31 to 1.52)***		18.3	17.3	1.06 (0.95 to 1.19)
Boxing Day	61.1	50.3	1.21 (1.14 to 1.3)***		41.2	33	1.25 (1.15 to 1.35)***		19.9	17.3	1.15 (1.03 to 1.29)
New Year’s Eve	46.3	50.3	0.92 (0.86 to 0.99)		31.1	33	0.94 (0.86 to 1.03)		15.2	17.3	0.88 (0.78 to 1)
New Year’s Day	60.4	50.3	1.2 (1.12 to 1.28)***		43.1	33	1.3 (1.21 to 1.41)***		17.3	17.3	1 (0.89 to 1.13)
Epiphany	46	50.3	0.92 (0.85 to 0.99)		29.9	33	0.91 (0.83 to 0.99)		16.1	17.3	0.93 (0.82 to 1.06)
**Easter**	52.5	50.3	1.04 (1.01 to 1.08)		35	33.6	1.04 (1 to 1.09)		17.5	16.7	1.05 (0.99 to 1.12)
Good Friday	50.9	50.3	1.01 (0.94 to 1.09)		32.1	33.6	0.96 (0.88 to 1.04)		18.8	16.7	1.13 (1.01 to 1.27)
Easter Eve	51.9	50.3	1.03 (0.96 to 1.11)		35.3	33.6	1.05 (0.97 to 1.14)		16.6	16.7	1 (0.88 to 1.13)
Easter Sunday	55.1	50.3	1.1 (1.03 to 1.17)		37.1	33.6	1.11 (1.02 to 1.2)		18	16.7	1.08 (0.96 to 1.21)
Easter Monday	52.2	50.3	1.04 (0.97 to 1.11)		35.6	33.6	1.06 (0.97 to 1.15)		16.6	16.7	0.99 (0.88 to 1.12)
**Midsummer**	51.9	46.2	1.12 (1.07 to 1.18)**		35.3	30.4	1.16 (1.09 to 1.23)**		16.6	15.8	1.05 (0.96 to 1.15)
Sports events											
UEFA Euro Cup	47.3	46.9	1.01 (0.97 to 1.04)		31	31.1	1 (0.95 to 1.04)		16.3	15.8	1.03 (0.97 to 1.09)
FIFA World Cup	45.2	46.2	0.98 (0.95 to 1.01)		29.8	30.7	0.97 (0.93 to 1.01)		15.4	15.5	0.99 (0.94 to 1.05)
Winter Olympic Games	48.2	50	0.96 (0.92 to 1.01)		31.6	32.7	0.97 (0.92 to 1.02)		16.6	17.3	0.96 (0.89 to 1.03)
Summer Olympic Games	47.4	45.6	1.04 (1 to 1.08)		31.6	30.4	1.04 (0.99 to 1.1)		15.8	15.2	1.04 (0.96 to 1.12)

P values are adjusted for multiple testing.

IRR=incidence rate ratio.

* =P<0.05.

** P<0.01

*** P<0.001

### Sports events

During the study period, four FIFA World Cup tournaments and four UEA European Championships took place. Sports events were not associated with a higher incidence of myocardial infarction, although we observed a trend for summer Olympic Games in men, but this did not reach statistical significance after adjusting for multiple testing (table 1, supplementary table 2).

### Time aspects

**Fig 3:**
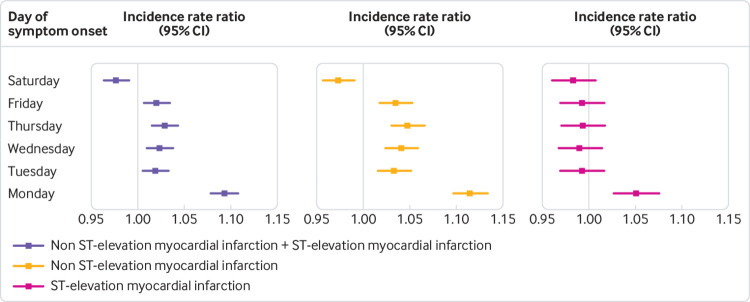
Results of day of week of onset of myocardial infarction symptoms. Shown are the associated risks of overall myocardial infarction, ST elevation myocardial infarction, and non-ST elevation myocardial infarction expressed as incidence rate ratios for days of the week. The reference period for day of week is Sunday

**Fig 4:**
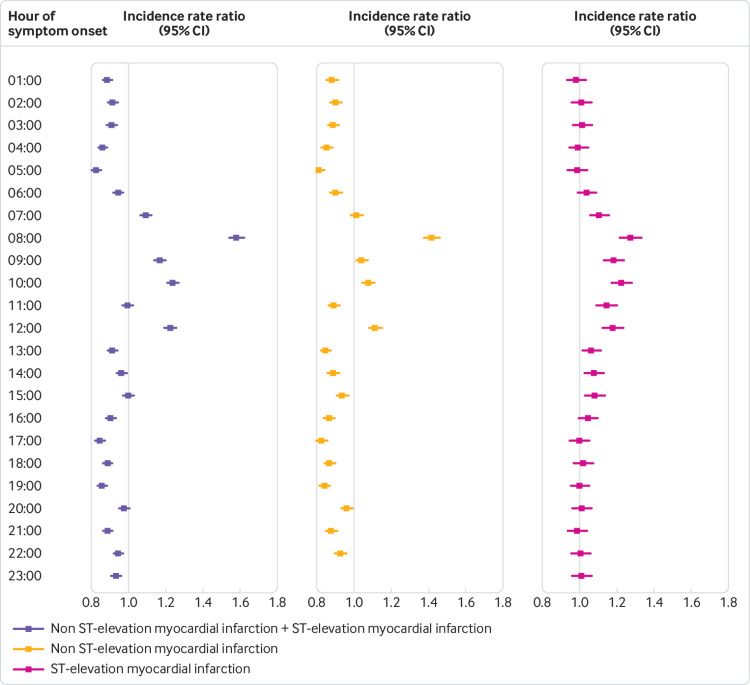
Results of hour of symptom onset. Reference hour for hour of symptom onset is 12 am Hours are defined as the hour of symptom onset, ie, 12 am-12 59 am P values after adjustment for multiple testing are shown in supplementary table 1

A statistically significant circaseptan variation in the incidence of myocardial infarction was evident on Mondays, and after stratification into NSTEMI and STEMI (fig 3). We observed a statistically significantly higher incidence of myocardial infarction in the analysis of hour of symptom onset, peaking at 8 am, most prominently for NSTEMI (fig 3, fig 4, supplementary table 1).

### Subgroup, sensitivity, and post hoc analyses

**Fig 5:**
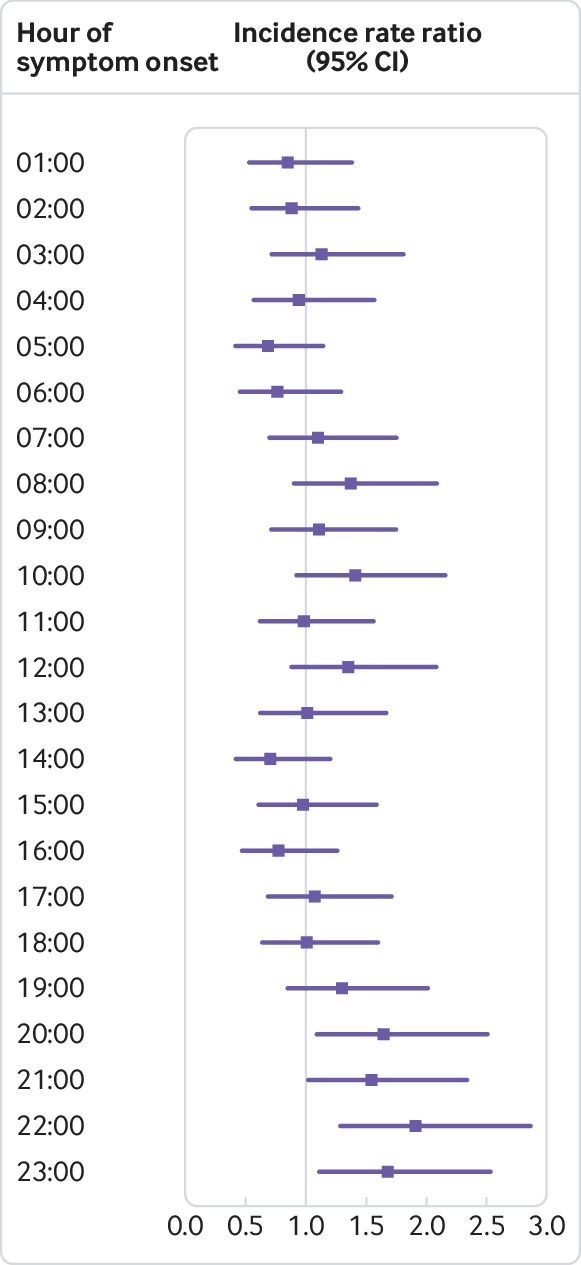
Hour of symptom onset on Christmas Eve. Shown are the associated risks of myocardial infarction expressed as incidence rate ratios for the hours of Christmas Eve. The reference hour is 12 am Hours are defined as the hour of symptom onset, ie, 12 am-12 59 am

We observed consistent results for all subgroups, with a higher incidence rate ratio of myocardial infarction during the Christmas holiday except for the subgroup of current smokers (supplementary fig 1, supplementary table 2). The risk was higher in patients aged ≥75, patients with diabetes, and those with a history of coronary artery disease. No subgroup was particularly more affected at Easter or Midsummer. We saw consistent results with higher risk of myocardial infarction on Mondays in all subgroups except for current smokers (supplementary fig 3, supplementary table 2). We observed consistent results for hour of symptom onset in all subgroups (supplementary fig 4, supplementary table 2). We conducted an analysis of Christmas and Midsummer holidays for all patients who had undergone coronary angiography with subsequent revascularisation with percutaneous coronary intervention or coronary artery bypass grafting versus patients who had undergone coronary angiography with no revascularisation. The associated higher risk of myocardial infarction during the holidays was limited to patients with myocardial infarction who had not undergone revascularisation (supplementary fig 2). We observed no associated risk in the sensitivity analyses exploring only the days when Sweden played in the FIFA World Cup or UEFA European Championship. The sensitivity analyses controlling for long term trends in myocardial infarction resulted in consistent results. We conducted a post hoc analysis of hour of symptom onset on Christmas Eve to investigate whether the altered risk associated with this holiday is also reflected by an altered circadian variation in myocardial infarction. Whereas most previous studies have shown an early morning peak in myocardial infarction; on Christmas Eve the peak was at 10 pm (fig 5).

## Discussion

We investigated the risk of myocardial infarction during national holidays, sports events, and various time periods using data on date and time of symptom onset, documented to the nearest minute in a large nationwide setting with 16 years of data on myocardial infarction. We observed a higher risk of myocardial infarction during Christmas/New Year and Midsummer holidays, but not at Easter. Sports events were not associated with higher risk of myocardial infarction. We observed a prominent peak incidence on calendar week 52, Mondays, and at 8 am. This is to our knowledge the largest study utilising electrocardiographic and biomarker positive myocardial infarction from a well known registry. These results are in line with previous studies using administrative data.

### Major holidays

We observed the highest risk of myocardial infarction (37% higher than during the control period) on Christmas Eve. In addition, myocardial infarctions on Christmas Eve peaked at around 10 pm, rather than in the early morning hours. Previous meta-analyses have shown that acute experience of anger, anxiety, sadness, grief, and stress increases the risk of myocardial infarction and thus possibly explains the higher risk observed in our study.^8^ ^13^ The association of higher risk at Christmas was more pronounced in people older than 75, those with known diabetes, and those with a history of coronary artery disease. These findings warrant further research to identify the mechanisms behind this phenomenon. Understanding what factors, activities, and emotions precede these myocardial infarctions and how they differ from myocardial infarctions experienced on other days could help develop a strategy to manage and reduce the number of these events. It is possible that family members visiting relatives after a long time apart find them in a poor general condition and decide to admit them to hospital. If this were the case, we would expect to see a decline in the number of myocardial infarctions in the weeks after Christmas compared with the weeks leading up to the holiday. Similarly, patients might delay reporting symptoms and seeking care because of unwillingness to disrupt the celebrations, and we would expect this to result in lower rates of myocardial infarction before Christmas than afterwards. However, the absence of any decline preceding or following Christmas indicates that these behavioural aspects are not the main contributing factors to the Christmas peak in myocardial infarction.

We observed a 20% higher risk of myocardial infarction on New Year’s Day. This could be due to the effects of excess alcohol and food consumption, exposure to cold temperatures at night, or sleep deprivation on New Year’s Eve. The associated risk of myocardial infarction during all holidays was similar between men and women, except for Midsummer, which was associated with a trend towards higher risk in men. It is possible that men are more likely to smoke, consume alcohol, and eat to excess during this holiday than women. Although no sex specific statistics are available to support this, statistics on the sale of alcohol from a government owned monopoly chain of retail stores shows sales peaking at Christmas and Midsummer.^27^

### Time aspects

We confirm previous studies that use administrative data and circadian and circaseptan variation in myocardial infarction.^4^ ^6^ ^28^ ^29^ The incidence of myocardial infarction peaked on calendar week 52, on Mondays, and at around 8 am. The rate of STEMI had a normal, lightly skewed distribution, whereas NSTEMI ran a more fluctuating course throughout the day (fig 5). Mondays were associated with the highest risk of myocardial infarction and we observed differences between STEMI and NSTEMI. Risk of NSTEMI was higher on weekdays than weekends, but no other day of week was associated with higher or lower risk of STEMI. To rule out behavioural factors, such as delay in patients seeking care, we used symptom onset and not admission date. Patient delay in seeking medical attention may still confound the results, however, as symptom onset may be less defined in patients with NSTEMI. The decline in incidence of NSTEMI at weekends and at night is supportive of this. By contrast, STEMI usually presents with more pronounced symptoms and is usually treated with minimal delay. Previous studies have shown a higher risk of myocardial infarction in the working population.^29^ By contrast, we found the pattern to be similar in both retired (≥75 years) and younger patients (<75 years). Previously proposed explanations to the circaseptan peak in myocardial infarction include stressful Mondays and a rise in arterial blood pressure and heart rate.^30^ The circadian variation has been attributed to peak cortisol levels, increased blood viscosity, and platelet aggregability in addition to a rise in arterial blood pressure and heart rate in the morning hours.^5^

### Sports events

Sports events were not associated with a higher risk of myocardial infarction, which was contrary to our expectations based on previous studies.^17^ ^31^ Wilbert-Lampen et al. presented an increased incidence in myocardial infarction in the Greater München area during the 2006 FIFA World Cup in Germany. Several aspects may contribute to the discrepancy in results. Germany was the host nation for the tournament, which might infer involvement by people who do not habitually follow football, resulting in a greater exposure to the sports event. In addition, the associated increased risk was restricted to days where the German football team was involved in the tournament, and highest during the days of the quarter and semi finals. The risk was neutralised on the day the German team played for third place. The only day associated with a higher risk and which did not involve the German team was the final, played between France and Italy. Together, these factors indicate that a strong emotional stress may be required to trigger myocardial infarction. We tried to address this in our sensitivity analysis of days where the Swedish team was playing. Our analysis did not show any associated risk. Moreover, no subgroup experienced a higher risk of myocardial infarction during any sport periods studied. Viewing sports events can therefore be considered safe.

### Pathophysiological aspects

We were able to characterise myocardial infarction to a higher degree than in previous studies and this enabled us to study STEMI and NSTEMI independently, together with a wide range of subgroups. Our results showed a consistently higher risk of myocardial infarction mainly due to higher rates of NSTEMI and a greater number of patients who were elderly, had diabetes, a history of coronary artery disease, or were already taking other medication. This indicates that the “vulnerable patient,” who may have risk factors such as blood vulnerable to thrombosis and myocardium vulnerable to arrhythmias in addition to vulnerable plaques, may be more prone to these precipitators of disease.^32^

We cannot rule out the suggestion that activities and emotions associated with holidays may result in myocardial infarction secondary to ischaemia, due to an increased oxygen demand in older and sicker patients. This is supported by the subgroup analysis on the Christmas and New Year’s holiday (supplementary fig 2) that showed an incremental risk increase of myocardial infarction with each age quartile.^33^ Infarct type classification was not introduced in the SWEDEHEART registry until 2010, therefore, in order to explore this area we conducted a post hoc analysis of the risk of myocardial infarction resulting in a coronary angiography between 2004 and 2013. Neither Christmas nor Midsummer holiday were associated with risk of myocardial infarction that resulted in revascularisation; these holidays were rather associated with myocardial infarction in which percutaneous coronary intervention or surgery was not deemed necessary. This suggests that a large proportion of myocardial infarction with non-occlusive coronary arteries may account for the higher risk of myocardial infarction during these holidays. Although this post hoc analysis did not consider chronic total occlusions or distal occlusions that were left untreated by the interventionist, these findings warrant further investigation.

The rationale behind our subgroup analyses of patients using previous medications was to investigate the possible inhibitory mechanisms of certain pharmacotherapies on the short term triggers of myocardial infarction. Drugs lowering heart rate, reducing blood pressure, lipid levels, and platelet aggregation might reduce plaque vulnerability to external triggers. For example, β-adrenergic inhibitors have been shown to blunt the circadian variation in myocardial infarction by reducing heart rate and blood pressure and increasing coronary blood flow by prolonging diastole.^34^ However, in our study, patients using cardiovascular drugs had a similar or higher risk of myocardial infarction during holidays. Our explanation for this is therefore in line with our main theory—the medications are surrogate measures of a sicker population, more vulnerable to external triggers. However, in the subgroup analyses of circadian variation, the peak at 8 am was less prominent in patients on β -adrenergic inhibitors compared with patients without β -adrenergic inhibitors (supplementary fig 4), a finding in line with previous studies.

### Limitations

This is an observational study; therefore, causality cannot be determined as we cannot rule out unobserved confounders. Previous publications have shown a higher incidence of myocardial infarction when ambient temperatures are low. However, it is unlikely that national holidays should be associated with lower temperature to bias the results since we used control periods two weeks before and after the holidays.

### Conclusion

In this nationwide real world study covering 16 years of hospital admissions for myocardial infarction with symptom onset documented to the nearest minute, Christmas and Midsummer holidays were associated with a higher risk of myocardial infarction. Consistently, we observed a higher risk in older and sicker patients, suggesting a role of external triggers in vulnerable patients.

**What is already known on this topic**

In Western countries, cardiac mortality and hospital admission due to myocardial infarction has been observed to peak at the Christmas and New Year holiday

The risk of myocardial infarction has also been linked to football championships, hurricanes, and stock market crashes

It is therefore conjectured that factors associated with emotional stress, physical activity, and lifestyle changes may modulate the onset of myocardial infarction by acting as short term triggers

**What this study adds**

In this large study covering 16 years of clinical myocardial infarction data, a higher risk of myocardial infarction was observed during Christmas/New Year and Midsummer holidays but not during the Easter holiday

The highest risk was during Christmas Eve and in patients over 75, and those with previous diabetes and coronary artery disease

Sports events were not associated with higher risk of myocardial infarction

## Web Extra

Extra material supplied by authors

## Notes

Ethical approval: This study was approved by Lund University’s ethical committee (2015/297).

Data sharing: No additional data available.

Transparency: The lead authors (MAM, DE) affirm that this manuscript is an honest, accurate, and transparent account of the study being reported; that no important aspects of the study have been omitted; and that any discrepancies from the study as planned (and, if relevant, registered) have been explained.
