# Supplementary material for: Christmas, national holidays, sport events, and time factors as triggers of acute myocardial infarction: SWEDEHEART observational study 1998-2013
Source: BMJ. 2018 Dec 12;363:k4811. doi: 10.1136/bmj.k4811 (PMC6289164; doi:10.1136/bmj.k4811)
Supplement: Supplementary file 1 — Supplementary material: tables and figures [file mohm046569.ww1.pdf]

Supplementary Materials

Supplementary Table 1.

	All MIs		NSTEMI		STEMI	
Sunday	IRR (95%CI)	Q-value	IRR (95%CI)	Q-value	IRR (95%CI)	Q-value
Monday	1.09 (1.08-1.11)	<0.001	1.12 (1.1-1.13)	<0.001	1.05 (1.03-1.08)	0.024
Tuesday	1.02 (1.01-1.03)	1	1.03 (1.02-1.05)	0.07	0.99 (0.97-1.02)	0.993
Wednesday	1.02 (1.01-1.04)	0.327	1.04 (1.02-1.06)	0.002	0.99 (0.97-1.01)	0.993
Thursday	1.03 (1.02-1.04)	0.019	1.05 (1.03-1.07)	<0.001	0.99 (0.97-1.02)	0.993
Friday	1.02 (1.01-1.03)	1	1.03 (1.02-1.05)	0.002	0.99 (0.97-1.02)	0.993
Saturday	0.98 (0.96-0.99)	0.389	0.97 (0.96-0.99)	0.736	0.98 (0.96-1.01)	0.993
**Hour of symptom onset**						
01:00	0.89 (0.86-0.91)	<0.001	0.88 (0.85-0.91)	<0.001	0.98 (0.93-1.04)	0.993
02:00	0.92 (0.89-0.94)	<0.001	0.9 (0.87-0.93)	<0.001	1.01 (0.96-1.07)	0.993
03:00	0.91 (0.88-0.94)	<0.001	0.89 (0.86-0.92)	<0.001	1.02 (0.96-1.07)	0.993
04:00	0.86 (0.84-0.89)	<0.001	0.85 (0.83-0.88)	<0.001	0.99 (0.94-1.05)	0.993
05:00	0.83 (0.8-0.85)	<0.001	0.81 (0.78-0.84)	<0.001	0.99 (0.94-1.04)	0.993
06:00	0.95 (0.92-0.97)	0.042	0.9 (0.87-0.93)	<0.001	1.04 (0.99-1.09)	0.993
07:00	1.09 (1.07-1.12)	<0.001	1.01 (0.98-1.05)	0.998	1.11 (1.05-1.16)	0.023
08:00	1.58 (1.55-1.62)	<0.001	1.42 (1.38-1.46)	<0.001	1.28 (1.22-1.34)	<0.001
09:00	1.17 (1.14-1.2)	<0.001	1.04 (1.01-1.07)	0.998	1.18 (1.13-1.24)	<0.001
10:00	1.24 (1.21-1.27)	<0.001	1.08 (1.04-1.11)	0.001	1.23 (1.17-1.28)	<0.001
11:00	1 (0.97-1.02)	1	0.89 (0.86-0.92)	<0.001	1.15 (1.1-1.2)	<0.001
12:00	1.23 (1.2-1.26)	<0.001	1.11 (1.08-1.15)	<0.001	1.18 (1.13-1.24)	<0.001
13:00	0.91 (0.89-0.94)	<0.001	0.85 (0.82-0.88)	<0.001	1.06 (1.01-1.12)	0.993
14:00	0.96 (0.93-0.99)	1	0.89 (0.86-0.92)	<0.001	1.08 (1.03-1.13)	0.993
15:00	1 (0.97-1.03)	1	0.94 (0.91-0.97)	0.042	1.08 (1.03-1.14)	0.745
16:00	0.9 (0.88-0.93)	<0.001	0.87 (0.84-0.9)	<0.001	1.05 (1-1.1)	0.993
17:00	0.85 (0.82-0.87)	<0.001	0.83 (0.8-0.86)	<0.001	1 (0.95-1.05)	0.993
18:00	0.89 (0.86-0.91)	<0.001	0.87 (0.84-0.9)	<0.001	1.02 (0.97-1.07)	0.993
19:00	0.86 (0.83-0.88)	<0.001	0.84 (0.81-0.87)	<0.001	1 (0.95-1.05)	0.993
20:00	0.98 (0.95-1)	1	0.96 (0.93-0.99)	0.998	1.01 (0.96-1.07)	0.993
21:00	0.89 (0.86-0.91)	<0.001	0.88 (0.85-0.91)	<0.001	0.99 (0.94-1.04)	0.993
22:00	0.95 (0.92-0.97)	0.036	0.93 (0.9-0.96)	0.004	1.01 (0.96-1.06)	0.993
23:00	0.93 (0.91-0.96)	0.001	0.92 (0.89-0.95)	<0.001	1.01 (0.96-1.07)	0.993

Supplementary Table 1. Results of circadian and circaseptan variation.
All P-values are adjusted for multiple testing. Hours are defined as the hour of symptom onset. i.e. 00:00 - 00:59. Sunday and midnight were set as reference periods.

**Supplementary Table 2.**	Total MI		Non ST-Elevation MI		ST-Elevation MI	
Christmas/New Years	IRR (95%CI)	Q-value	IRR (95%CI)	Q-value	IRR (95%CI)	Q-value
Men	1.15 (1.11-1.19)	<0.001	1.22 (1.16-1.27)	<0.001	1.02 (0.96-1.09)	0.993
Women	1.16 (1.11-1.22)	<0.001	1.21 (1.14-1.29)	<0.001	1.06 (0.97-1.16)	0.993
Age≥75yo	1.22 (1.17-1.27)	<0.001	1.28 (1.21-1.34)	<0.001	1.06 (0.98-1.16)	0.993
Age<75yo	1.1 (1.05-1.14)	0.009	1.15 (1.09-1.21)	<0.001	1.01 (0.95-1.09)	0.993
Current Smoker	1.09 (1.02-1.17)	1.000	1.13 (1.03-1.24)	0.998	1.05 (0.94-1.16)	0.993
Non Smoker	1.16 (1.12-1.2)	<0.001	1.18 (1.13-1.22)	<0.001	1.12 (1.08-1.17)	<0.001
Diabetes	1.28 (1.2-1.36)	<0.001	1.33 (1.24-1.42)	<0.001	1.12 (0.99-1.27)	0.993
No Diabetes	1.11 (1.08-1.15)	<0.001	1.18 (1.13-1.23)	<0.001	1.01 (0.95-1.07)	0.993
Hypertension	1.16 (1.11-1.22)	<0.001	1.21 (1.15-1.28)	<0.001	1.05 (0.96-1.14)	0.993
No Hypertension	1.13 (1.09-1.18)	<0.001	1.21 (1.15-1.27)	<0.001	1.01 (0.95-1.09)	0.993
CAD	1.26 (1.2-1.32)	<0.001	1.26 (1.2-1.32)	<0.001	1.26 (1.2-1.32)	<0.001
No CAD	1.09 (1.05-1.14)	0.002	1.15 (1.1-1.21)	<0.001	1 (0.94-1.07)	0.993
Beta-blockers	1.21 (1.16-1.27)	<0.001	1.27 (1.21-1.33)	<0.001	1.06 (0.96-1.16)	0.993
No Beta-blockers	1.1 (1.06-1.15)	0.001	1.16 (1.1-1.22)	<0.001	1.02 (0.96-1.09)	0.993
Ca-inhibitors	1.24 (1.16-1.33)	<0.001	1.3 (1.2-1.41)	<0.001	1.07 (0.93-1.23)	0.993
No Ca-inhibitors	1.13 (1.1-1.17)	<0.001	1.19 (1.14-1.24)	<0.001	1.03 (0.97-1.09)	0.993
Aspirin	1.22 (1.16-1.27)	<0.001	1.26 (1.2-1.33)	<0.001	1.07 (0.98-1.18)	0.993
No Aspirin	1.1 (1.06-1.15)	0.002	1.17 (1.11-1.23)	<0.001	1.01 (0.95-1.08)	0.993
ACE-I/ARB	1.15 (1.09-1.21)	<0.001	1.16 (1.1-1.22)	<0.001	1.19 (1.12-1.26)	<0.001
No ACE-I/ARB	1.16 (1.12-1.2)	<0.001	1.22 (1.15-1.29)	<0.001	0.91 (0.84-0.99)	0.993
Statins	1.19 (1.12-1.25)	<0.001	1.26 (1.18-1.34)	<0.001	0.95 (0.84-1.08)	0.993
No Statins	1.14 (1.1-1.18)	<0.001	1.19 (1.14-1.25)	<0.001	1.05 (0.99-1.12)	0.993
Easter						
Men	1.04 (1-1.09)	1.000	1.04 (0.98-1.1)	0.998	1.05 (0.97-1.13)	0.993
Women	1.05 (0.99-1.11)	1.000	1.05 (0.98-1.13)	0.998	1.05 (0.94-1.17)	0.993
Age≥75yo	1.07 (1.02-1.13)	1.000	1.08 (1.02-1.15)	0.998	1.05 (0.95-1.17)	0.993
Age<75yo	1.02 (0.97-1.07)	1.000	1 (0.94-1.07)	0.998	1.05 (0.97-1.13)	0.993
Current Smoker	0.99 (0.91-1.07)	1.000	0.97 (0.87-1.09)	0.998	1.01 (0.89-1.14)	0.993
Non Smoker	1.07 (1.03-1.12)	0.422	1.07 (1.02-1.12)	0.998	1.07 (1.01-1.12)	0.993
Diabetes	1.11 (1.03-1.2)	1.000	1.15 (1.06-1.26)	0.490	0.99 (0.84-1.16)	0.993
No Diabetes	1.03 (0.99-1.07)	1.000	1.01 (0.96-1.06)	0.998	1.06 (0.99-1.14)	0.993
Hypertension	1.08 (1.02-1.14)	1.000	1.08 (1.01-1.15)	0.998	1.09 (0.98-1.2)	0.993
No Hypertension	1.02 (0.97-1.07)	1.000	1.02 (0.96-1.09)	0.998	1.03 (0.95-1.11)	0.993
CAD	1.11 (1.04-1.18)	0.327	1.11 (1.04-1.18)	0.296	1.11 (1.04-1.18)	0.889
No CAD	1.01 (0.97-1.06)	1.000	0.99 (0.93-1.05)	0.998	1.03 (0.96-1.11)	0.993
Beta-blockers	1.08 (1.02-1.14)	1.000	1.09 (1.02-1.16)	0.998	1.06 (0.95-1.19)	0.993
No Beta-blockers	1.02 (0.97-1.07)	1.000	1 (0.94-1.07)	0.998	1.04 (0.97-1.13)	0.993
Ca-inhibitors	1.13 (1.04-1.23)	1.000	1.11 (1.01-1.23)	0.998	1.19 (1.01-1.4)	0.993
No Ca-inhibitors	1.03 (0.99-1.07)	1.000	1.03 (0.98-1.08)	0.998	1.03 (0.96-1.1)	0.993

Aspirin	1.07 (1.01-1.13)	1.000	1.06 (1-1.13)	0.998	1.07 (0.96-1.2)	0.993
No Aspirin	1.03 (0.98-1.08)	1.000	1.02 (0.96-1.09)	0.998	1.04 (0.96-1.12)	0.993
ACE-I/ARB	1.1 (1.03-1.17)	1.000	1.1 (1.03-1.17)	0.998	1.11 (1.03-1.19)	0.993
No ACE-I/ARB	1.02 (0.98-1.07)	1.000	1.04 (0.97-1.12)	0.998	1.04 (0.95-1.15)	0.993
Statins	1.04 (0.97-1.12)	1.000	1.07 (0.98-1.15)	0.998	0.95 (0.81-1.11)	0.993
No Statins	1.05 (1-1.09)	1.000	1.03 (0.98-1.09)	0.998	1.07 (1-1.15)	0.993
Midsummer						
Men	1.15 (1.08-1.23)	0.003	1.21 (1.12-1.3)	0.001	1.06 (0.95-1.18)	0.993
Women	1.07 (0.98-1.16)	1.000	1.09 (0.98-1.2)	0.998	1.03 (0.89-1.2)	0.993
Age≥75yo	1.14 (1.06-1.22)	0.246	1.18 (1.08-1.29)	0.049	1.02 (0.88-1.18)	0.993
Age<75yo	1.11 (1.04-1.19)	0.726	1.14 (1.05-1.25)	0.814	1.07 (0.95-1.19)	0.993
Current Smoker	1.03 (0.91-1.15)	1.000	1.11 (0.95-1.29)	0.998	0.93 (0.77-1.11)	0.993
Non Smoker	1.14 (1.08-1.21)	0.005	1.16 (1.09-1.24)	0.001	1.11 (1.04-1.19)	0.993
Diabetes	1.19 (1.07-1.32)	0.454	1.22 (1.09-1.38)	0.330	1.08 (0.87-1.34)	0.993
No Diabetes	1.11 (1.05-1.17)	0.196	1.14 (1.07-1.23)	0.078	1.04 (0.95-1.15)	0.993
Hypertension	1.18 (1.1-1.28)	0.004	1.23 (1.12-1.34)	0.003	1.09 (0.95-1.25)	0.993
No Hypertension	1.08 (1.01-1.16)	1.000	1.11 (1.02-1.21)	0.998	1.03 (0.92-1.15)	0.993
CAD	1.19 (1.1-1.29)	0.010	1.19 (1.09-1.29)	0.018	1.19 (1.1-1.29)	0.028
No CAD	1.09 (1.02-1.16)	1.000	1.13 (1.04-1.23)	0.998	1.02 (0.92-1.13)	0.993
Beta-blockers	1.21 (1.12-1.3)	<0.001	1.27 (1.17-1.38)	<0.001	1.03 (0.88-1.21)	0.993
No Beta-blockers	1.06 (0.99-1.14)	1.000	1.06 (0.98-1.16)	0.998	1.06 (0.95-1.18)	0.993
Ca-inhibitors	1.18 (1.05-1.32)	1.000	1.29 (1.13-1.47)	0.065	0.89 (0.7-1.15)	0.993
No Ca-inhibitors	1.11 (1.05-1.18)	0.070	1.13 (1.06-1.21)	0.144	1.08 (0.98-1.18)	0.993
Aspirin	1.23 (1.14-1.32)	<0.001	1.28 (1.18-1.39)	<0.001	1.07 (0.91-1.25)	0.993
No Aspirin	1.04 (0.97-1.12)	1.000	1.04 (0.95-1.14)	0.998	1.04 (0.94-1.16)	0.993
ACE-I/ARB	1.15 (1.06-1.26)	0.515	1.17 (1.07-1.28)	0.288	1.15 (1.04-1.27)	0.993
No ACE-I/ARB	1.11 (1.04-1.18)	0.277	1.14 (1.04-1.26)	0.998	1.06 (0.93-1.21)	0.993
Statins	1.23 (1.12-1.35)	0.009	1.24 (1.12-1.38)	0.022	1.17 (0.95-1.42)	0.993
No Statins	1.09 (1.02-1.15)	1.000	1.13 (1.04-1.21)	0.590	1.03 (0.93-1.13)	0.993
UEFA Euro Cup						
Men	0.99 (0.95-1.04)	1.000	0.97 (0.92-1.03)	0.998	1.04 (0.96-1.12)	0.993
Women	1.03 (0.97-1.09)	1.000	1.04 (0.97-1.12)	0.998	1.01 (0.91-1.13)	0.993
Age≥75yo	1.01 (0.96-1.06)	1.000	1.01 (0.95-1.08)	0.998	1 (0.91-1.11)	0.993
Age<75yo	1.01 (0.96-1.06)	1.000	0.98 (0.92-1.05)	0.998	1.05 (0.97-1.13)	0.993
Current Smoker	1.03 (0.95-1.11)	1.000	1.03 (0.92-1.14)	0.998	1.03 (0.91-1.16)	0.993
Non Smoker	0.99 (0.95-1.04)	1.000	0.98 (0.94-1.03)	0.998	1.01 (0.96-1.06)	0.993
Diabetes	1 (0.93-1.08)	1.000	0.95 (0.87-1.04)	0.998	1.15 (1-1.34)	0.993
No Diabetes	1.01 (0.97-1.05)	1.000	1.01 (0.96-1.07)	0.998	1.01 (0.94-1.08)	0.993
Hypertension	1.01 (0.95-1.06)	1.000	0.98 (0.92-1.04)	0.998	1.08 (0.98-1.19)	0.993
No Hypertension	1.01 (0.96-1.06)	1.000	1.02 (0.96-1.08)	0.998	1 (0.92-1.09)	0.993
CAD	1.02 (0.96-1.08)	1.000	1.02 (0.96-1.08)	0.998	1.01 (0.95-1.07)	0.993
No CAD	1 (0.96-1.05)	1.000	0.99 (0.93-1.05)	0.998	1.02 (0.95-1.09)	0.993
Beta-blockers	1.01 (0.96-1.07)	1.000	0.98 (0.92-1.05)	0.998	1.09 (0.98-1.22)	0.993
No Beta-blockers	1 (0.96-1.05)	1.000	1.01 (0.95-1.07)	0.998	1 (0.93-1.08)	0.993
Ca-inhibitors	0.99 (0.91-1.08)	1.000	0.99 (0.89-1.09)	0.998	0.99 (0.84-1.16)	0.993
No Ca-inhibitors	1.01 (0.97-1.05)	1.000	1 (0.95-1.05)	0.998	1.04 (0.97-1.11)	0.993
Aspirin	1 (0.95-1.06)	1.000	1 (0.94-1.06)	0.998	1.01 (0.91-1.13)	0.993

No Aspirin	1.01 (0.97-1.06)	1.000	1 (0.93-1.06)	0.998	1.04 (0.96-1.12)	0.993
ACE-I/ARB	0.99 (0.93-1.06)	1.000	0.98 (0.92-1.05)	0.998	0.99 (0.93-1.07)	0.993
No ACE-I/ARB	1.02 (0.97-1.06)	1.000	1.19 (1.11-1.27)	<0.001	1.26 (1.16-1.38)	<0.001
Statins	1 (0.93-1.07)	1.000	0.98 (0.91-1.06)	0.998	1.07 (0.93-1.24)	0.993
No Statins	1.01 (0.97-1.05)	1.000	1 (0.95-1.06)	0.998	1.02 (0.95-1.09)	0.993
FIFA world cup						
Men	0.99 (0.95-1.03)	1.000	0.98 (0.93-1.03)	0.998	1.01 (0.94-1.08)	0.993
Women	0.96 (0.91-1.01)	1.000	0.96 (0.9-1.02)	0.998	0.96 (0.87-1.06)	0.993
Age≥75yo	0.95 (0.91-1)	1.000	0.95 (0.9-1.01)	0.998	0.96 (0.88-1.05)	0.993
Age<75yo	1 (0.95-1.04)	1.000	0.99 (0.94-1.05)	0.998	1.01 (0.94-1.08)	0.993
Current Smoker	0.93 (0.86-1)	1.000	0.94 (0.85-1.03)	0.998	0.92 (0.83-1.03)	0.993
Non Smoker	1 (0.96-1.03)	1.000	1 (0.96-1.04)	0.998	1.03 (0.98-1.08)	0.993
Diabetes	0.96 (0.9-1.03)	1.000	0.95 (0.87-1.03)	0.998	1.01 (0.88-1.16)	0.993
No Diabetes	0.98 (0.94-1.02)	1.000	0.98 (0.93-1.02)	0.998	0.99 (0.93-1.05)	0.993
Hypertension	0.94 (0.9-0.99)	1.000	0.93 (0.88-0.99)	0.998	0.96 (0.88-1.06)	0.993
No Hypertension	1.01 (0.96-1.05)	1.000	1 (0.95-1.06)	0.998	1.01 (0.94-1.09)	0.993
CAD	0.99 (0.94-1.05)	1.000	1 (0.95-1.05)	0.998	0.99 (0.94-1.05)	0.993
No CAD	0.97 (0.93-1.01)	1.000	0.96 (0.91-1.01)	0.998	0.96 (0.9-1.03)	0.993
Beta-blockers	0.95 (0.9-1)	1.000	0.94 (0.89-1)	0.998	0.97 (0.87-1.07)	0.993
No Beta-blockers	1 (0.96-1.04)	1.000	1 (0.94-1.05)	0.998	1 (0.94-1.07)	0.993
Ca-inhibitors	1.01 (0.93-1.09)	1.000	1.01 (0.92-1.11)	0.998	0.99 (0.86-1.15)	0.993
No Ca-inhibitors	0.97 (0.94-1.01)	1.000	0.96 (0.92-1.01)	0.998	0.99 (0.93-1.05)	0.993
Aspirin	0.95 (0.9-1)	1.000	0.95 (0.9-1)	0.998	0.95 (0.86-1.05)	0.993
No Aspirin	1 (0.96-1.05)	1.000	1 (0.94-1.05)	0.998	1.01 (0.94-1.08)	0.993
ACE-I/ARB	0.91 (0.85-0.96)	0.486	0.91 (0.86-0.97)	0.910	0.92 (0.86-0.99)	0.993
No ACE-I/ARB	1.01 (0.97-1.05)	1.000	0.88 (0.82-0.94)	0.093	0.89 (0.81-0.97)	0.993
Statins	0.9 (0.85-0.97)	0.884	0.9 (0.84-0.97)	0.998	0.92 (0.81-1.06)	0.993
No Statins	1 (0.97-1.04)	1.000	1 (0.96-1.05)	0.998	1 (0.95-1.07)	0.993
Winter Olympic Games						
Men	0.99 (0.94-1.04)	1.000	1 (0.94-1.07)	0.998	0.96 (0.88-1.05)	0.993
Women	0.92 (0.86-0.99)	1.000	0.91 (0.83-0.99)	0.998	0.94 (0.83-1.07)	0.993
Age≥75yo	0.98 (0.92-1.04)	1.000	0.96 (0.89-1.04)	0.998	1.01 (0.9-1.14)	0.993
Age<75yo	0.95 (0.9-1.01)	1.000	0.97 (0.9-1.05)	0.998	0.92 (0.84-1.01)	0.993
Current Smoker	0.95 (0.86-1.05)	1.000	1.04 (0.91-1.18)	0.998	0.85 (0.73-0.98)	0.993
Non Smoker	0.97 (0.92-1.02)	1.000	0.97 (0.92-1.03)	0.998	0.97 (0.91-1.03)	0.993
Diabetes	0.9 (0.82-0.98)	1.000	0.92 (0.83-1.03)	0.998	0.82 (0.69-0.99)	0.993
No Diabetes	0.98 (0.93-1.03)	1.000	0.98 (0.92-1.04)	0.998	0.98 (0.91-1.06)	0.993
Hypertension	0.91 (0.86-0.98)	1.000	0.89 (0.82-0.96)	0.998	0.97 (0.86-1.09)	0.993
No Hypertension	1 (0.94-1.06)	1.000	1.03 (0.96-1.11)	0.998	0.95 (0.86-1.04)	0.993
CAD	1.01 (0.94-1.09)	1.000	1.02 (0.95-1.09)	0.998	1.02 (0.95-1.1)	0.993
No CAD	0.93 (0.89-0.99)	1.000	0.94 (0.87-1.01)	0.998	0.94 (0.86-1.02)	0.993
Beta-blockers	0.96 (0.9-1.03)	1.000	0.96 (0.89-1.04)	0.998	0.96 (0.85-1.1)	0.993
No Beta-blockers	0.96 (0.91-1.02)	1.000	0.97 (0.9-1.05)	0.998	0.95 (0.87-1.04)	0.993
Ca-inhibitors	0.88 (0.79-0.97)	1.000	0.88 (0.78-1)	0.998	0.86 (0.7-1.05)	0.993
No Ca-inhibitors	0.98 (0.94-1.03)	1.000	0.99 (0.93-1.05)	0.998	0.97 (0.9-1.05)	0.993

Aspirin	0.97 (0.91-1.03)	1.000	0.98 (0.91-1.06)	0.998	0.91 (0.8-1.04)	0.993
No Aspirin	0.96 (0.91-1.02)	1.000	0.95 (0.88-1.02)	0.998	0.97 (0.89-1.06)	0.993
ACE-I/ARB	0.9 (0.83-0.98)	1.000	0.89 (0.82-0.97)	0.998	0.93 (0.85-1.01)	0.993
No ACE-I/ARB	0.99 (0.94-1.04)	1.000	0.89 (0.81-0.97)	0.998	0.91 (0.81-1.02)	0.993
Statins	0.89 (0.82-0.97)	1.000	0.9 (0.82-1)	0.998	0.86 (0.73-1.03)	0.993
No Statins	0.99 (0.94-1.04)	1.000	0.99 (0.93-1.06)	0.998	0.98 (0.9-1.06)	0.993
Summer Olympic Games						
Men	1.06 (1.01-1.12)	1.000	1.09 (1.02-1.16)	0.998	1.02 (0.93-1.12)	0.993
Women	1 (0.93-1.07)	1.000	0.97 (0.89-1.06)	0.998	1.07 (0.94-1.21)	0.993
Age≥75yo	1.04 (0.98-1.11)	1.000	1.03 (0.95-1.1)	0.998	1.09 (0.97-1.23)	0.993
Age<75yo	1.04 (0.98-1.1)	1.000	1.06 (0.98-1.14)	0.998	1 (0.91-1.1)	0.993
Current Smoker	1.08 (0.99-1.19)	1.000	1.06 (0.93-1.2)	0.998	1.12 (0.97-1.3)	0.993
Non Smoker	1.02 (0.97-1.07)	1.000	1.02 (0.97-1.08)	0.998	1.01 (0.95-1.08)	0.993
Diabetes	1.04 (0.95-1.14)	1.000	1.01 (0.91-1.12)	0.998	1.14 (0.95-1.36)	0.993
No Diabetes	1.04 (0.99-1.09)	1.000	1.05 (0.99-1.12)	0.998	1.02 (0.94-1.1)	0.993
Hypertension	1.01 (0.94-1.07)	1.000	1 (0.93-1.08)	0.998	1.02 (0.91-1.15)	0.993
No Hypertension	1.06 (1-1.12)	1.000	1.06 (0.99-1.14)	0.998	1.06 (0.96-1.16)	0.993
CAD	1.04 (0.97-1.12)	1.000	1.04 (0.97-1.12)	0.998	1.03 (0.96-1.11)	0.993
No CAD	1.04 (0.99-1.1)	1.000	1.03 (0.96-1.1)	0.998	1.04 (0.96-1.13)	0.993
Beta-blockers	1.06 (0.99-1.13)	1.000	1.06 (0.99-1.15)	0.998	1.03 (0.9-1.17)	0.993
No Beta-blockers	1.03 (0.97-1.09)	1.000	1.02 (0.95-1.09)	0.998	1.04 (0.95-1.14)	0.993
Ca-inhibitors	1.1 (1-1.22)	1.000	1.13 (1-1.27)	0.998	1.05 (0.87-1.26)	0.993
No Ca-inhibitors	1.03 (0.98-1.08)	1.000	1.02 (0.96-1.08)	0.998	1.04 (0.96-1.12)	0.993
Aspirin	1.04 (0.97-1.11)	1.000	1.04 (0.96-1.12)	0.998	1.03 (0.9-1.18)	0.993
No Aspirin	1.04 (0.99-1.1)	1.000	1.04 (0.97-1.12)	0.998	1.04 (0.95-1.14)	0.993
ACE-I/ARB	1.01 (0.93-1.09)	1.000	0.98 (0.91-1.06)	0.998	0.99 (0.91-1.07)	0.993
No ACE-I/ARB	1.05 (1-1.11)	1.000	1.33 (1.23-1.44)	<0.001	1.28 (1.15-1.43)	0.003
Statins	1.06 (0.98-1.16)	1.000	1.06 (0.96-1.16)	0.998	1.09 (0.92-1.29)	0.993
No Statins	1.03 (0.98-1.08)	1.000	1.03 (0.97-1.1)	0.998	1.03 (0.94-1.11)	0.993
Men						
Monday	1.1 (1.08-1.12)	<0.001	1.13 (1.11-1.15)	<0.001	1.04 (1.01-1.07)	0.993
Tuesday	1.01 (1-1.03)	1.000	1.03 (1.01-1.05)	0.998	0.98 (0.95-1.01)	0.993
Wednesday	1.02 (1-1.03)	1.000	1.04 (1.02-1.06)	0.078	0.97 (0.95-1)	0.993
Thursday	1.02 (1-1.04)	1.000	1.05 (1.02-1.07)	0.016	0.98 (0.95-1.01)	0.993
Friday	1.01 (0.99-1.02)	1.000	1.03 (1-1.05)	0.078	0.97 (0.94-1)	0.993
Saturday	0.98 (0.96-0.99)	1.000	0.97 (0.95-0.99)	0.998	0.98 (0.95-1.01)	0.993
Women						
Monday	1.08 (1.06-1.11)	<0.001	1.09 (1.06-1.12)	<0.001	1.06 (1.02-1.11)	0.993
Tuesday	1.03 (1-1.05)	1.000	1.03 (1.01-1.06)	0.998	1.01 (0.97-1.06)	0.993
Wednesday	1.04 (1.01-1.06)	0.910	1.04 (1.01-1.07)	0.998	1.03 (0.98-1.07)	0.993
Thursday	1.04 (1.02-1.07)	0.147	1.05 (1.02-1.08)	0.181	1.03 (0.98-1.07)	0.993
Friday	1.04 (1.02-1.07)	0.083	1.05 (1.02-1.08)	0.998	1.04 (0.99-1.08)	0.993
Saturday	0.98 (0.96-1)	1.000	0.97 (0.95-1)	0.998	0.99 (0.95-1.03)	0.993
Age≥75yo						
Monday	1.1 (1.08-1.12)	<0.001	1.1 (1.07-1.13)	<0.001	1.1 (1.05-1.14)	0.003
Tuesday	1.04 (1.02-1.06)	0.036	1.03 (1.01-1.06)	0.998	1.06 (1.02-1.11)	0.993

Wednesday	1.07 (1.04-1.09)	<0.001	1.07 (1.04-1.09)	<0.001	1.06 (1.02-1.11)	0.993
Thursday	1.08 (1.05-1.1)	<0.001	1.08 (1.06-1.11)	<0.001	1.06 (1.02-1.1)	0.993
Friday	1.06 (1.04-1.08)	<0.001	1.07 (1.04-1.09)	<0.001	1.05 (1.01-1.1)	0.993
Saturday	0.98 (0.96-1)	1.000	0.98 (0.96-1.01)	0.998	0.99 (0.95-1.03)	0.993
Age<75yo						
Monday	1.09 (1.07-1.11)	<0.001	1.13 (1.1-1.16)	<0.001	1.03 (1-1.06)	0.993
Tuesday	1 (0.98-1.02)	1.000	1.03 (1.01-1.06)	0.998	0.95 (0.92-0.98)	0.960
Wednesday	0.99 (0.97-1.01)	1.000	1.02 (0.99-1.04)	0.998	0.95 (0.92-0.98)	0.425
Thursday	0.99 (0.97-1.01)	1.000	1.01 (0.99-1.04)	0.998	0.96 (0.93-0.98)	0.993
Friday	0.99 (0.97-1)	1.000	1 (0.98-1.03)	0.998	0.96 (0.93-0.99)	0.993
Saturday	0.97 (0.95-0.99)	0.820	0.96 (0.94-0.99)	0.998	0.98 (0.95-1.01)	0.993
Current Smoker						
Monday	1.04 (1.01-1.08)	1.000	1.07 (1.03-1.12)	0.287	1.01 (0.96-1.06)	0.993
Tuesday	0.98 (0.95-1.02)	1.000	1.01 (0.97-1.05)	0.998	0.95 (0.91-1)	0.993
Wednesday	1 (0.96-1.03)	1.000	1.04 (0.99-1.08)	0.998	0.95 (0.9-0.99)	0.993
Thursday	0.98 (0.95-1.01)	1.000	1.01 (0.97-1.06)	0.998	0.94 (0.89-0.98)	0.993
Friday	1 (0.97-1.04)	1.000	1.01 (0.97-1.06)	0.998	0.99 (0.95-1.04)	0.993
Saturday	0.98 (0.95-1.02)	1.000	0.98 (0.94-1.02)	0.998	0.99 (0.95-1.04)	0.993
Non Smoker						
Monday	1.11 (1.09-1.12)	<0.001	1.13 (1.11-1.15)	<0.001	1.06 (1.03-1.09)	0.066
Tuesday	1.02 (1.01-1.04)	1.000	1.03 (1.01-1.05)	0.415	1.01 (0.98-1.04)	0.993
Wednesday	1.03 (1.01-1.04)	0.406	1.04 (1.02-1.06)	0.066	1 (0.97-1.03)	0.993
Thursday	1.04 (1.02-1.05)	0.004	1.05 (1.03-1.07)	<0.001	1.01 (0.98-1.04)	0.993
Friday	1.02 (1-1.04)	1.000	1.04 (1.02-1.06)	0.066	0.99 (0.96-1.02)	0.993
Saturday	0.98 (0.96-0.99)	1.000	0.97 (0.95-0.99)	0.998	0.99 (0.96-1.01)	0.993
Diabetes						
Monday	1.09 (1.06-1.13)	<0.001	1.11 (1.07-1.15)	<0.001	1.05 (0.99-1.12)	0.993
Tuesday	1.06 (1.03-1.09)	0.073	1.06 (1.02-1.1)	0.384	1.06 (1-1.12)	0.993
Wednesday	1.03 (1-1.06)	1.000	1.03 (0.99-1.07)	0.998	1.03 (0.97-1.1)	0.993
Thursday	1.07 (1.04-1.1)	0.002	1.09 (1.05-1.13)	<0.001	1.02 (0.96-1.08)	0.993
Friday	1.07 (1.04-1.1)	0.003	1.08 (1.04-1.11)	0.998	1.05 (0.99-1.11)	0.993
Saturday	1 (0.97-1.03)	1.000	1.01 (0.98-1.05)	0.998	0.95 (0.89-1)	0.993
No Diabetes						
Monday	1.09 (1.08-1.11)	<0.001	1.12 (1.1-1.14)	<0.001	1.05 (1.02-1.08)	0.115
Tuesday	1.01 (0.99-1.02)	1.000	1.03 (1.01-1.05)	0.998	0.98 (0.95-1.01)	0.993
Wednesday	1.02 (1.01-1.04)	1.000	1.05 (1.03-1.07)	0.003	0.98 (0.96-1.01)	0.993
Thursday	1.02 (1-1.03)	1.000	1.03 (1.01-1.05)	0.332	0.99 (0.96-1.01)	0.993
Friday	1.01 (0.99-1.02)	1.000	1.02 (1-1.04)	0.003	0.98 (0.96-1.01)	0.993
Saturday	0.97 (0.96-0.99)	0.120	0.96 (0.94-0.98)	0.028	0.99 (0.96-1.02)	0.993
Hypertension						
Monday	1.09 (1.07-1.12)	<0.001	1.11 (1.08-1.14)	<0.001	1.06 (1.02-1.1)	0.993
Tuesday	1.04 (1.02-1.06)	0.045	1.04 (1.02-1.07)	0.420	1.04 (1-1.08)	0.993
Wednesday	1.04 (1.02-1.06)	0.190	1.04 (1.02-1.07)	0.221	1.02 (0.98-1.06)	0.993
Thursday	1.06 (1.04-1.09)	<0.001	1.07 (1.05-1.1)	<0.001	1.04 (1-1.08)	0.993
Friday	1.04 (1.02-1.06)	0.102	1.05 (1.02-1.08)	0.221	1.01 (0.98-1.05)	0.993
Saturday	0.99 (0.97-1.01)	1.000	0.99 (0.97-1.02)	0.998	0.99 (0.95-1.02)	0.993
No Hypertension						
Monday	1.09 (1.07-1.11)	<0.001	1.12 (1.1-1.15)	<0.001	1.05 (1.01-1.08)	0.993
Tuesday	1 (0.98-1.02)	1.000	1.03 (1-1.05)	0.998	0.96 (0.93-0.99)	0.993

Wednesday	1.01 (0.99-1.03)	1.000	1.04 (1.01-1.06)	0.527	0.97 (0.94-1)	0.993
Thursday	1 (0.99-1.02)	1.000	1.03 (1-1.05)	0.998	0.97 (0.94-1)	0.993
Friday	1.01 (0.99-1.02)	1.000	1.02 (1-1.05)	0.527	0.98 (0.95-1.01)	0.993
Saturday	0.97 (0.95-0.99)	0.329	0.96 (0.94-0.98)	0.243	0.98 (0.95-1.01)	0.993
CAD						
Monday	1.08 (1.05-1.1)	<0.001	1.08 (1.05-1.1)	<0.001	1.08 (1.03-1.14)	0.993
Tuesday	1.02 (0.99-1.04)	1.000	1.02 (1-1.05)	0.998	1 (0.95-1.05)	0.993
Wednesday	1.02 (1-1.05)	1.000	1.03 (1-1.05)	0.998	1.01 (0.96-1.06)	0.993
Thursday	1.04 (1.02-1.07)	0.210	1.05 (1.02-1.08)	0.155	1.02 (0.97-1.07)	0.993
Friday	1.04 (1.02-1.07)	0.168	1.05 (1.02-1.07)	0.998	1.03 (0.98-1.08)	0.993
Saturday	0.98 (0.96-1.01)	1.000	0.98 (0.96-1.01)	0.998	0.99 (0.94-1.04)	0.993
No CAD						
Monday	1.1 (1.08-1.12)	<0.001	1.14 (1.12-1.17)	<0.001	1.04 (1.02-1.07)	0.993
Tuesday	1.02 (1-1.04)	1.000	1.04 (1.02-1.06)	0.161	0.99 (0.96-1.02)	0.993
Wednesday	1.02 (1.01-1.04)	1.000	1.05 (1.03-1.08)	0.003	0.98 (0.96-1.01)	0.993
Thursday	1.02 (1-1.04)	1.000	1.05 (1.02-1.07)	0.019	0.99 (0.96-1.01)	0.993
Friday	1.01 (0.99-1.03)	1.000	1.03 (1-1.05)	0.003	0.98 (0.96-1.01)	0.993
Saturday	0.97 (0.96-0.99)	0.805	0.97 (0.95-0.99)	0.998	0.98 (0.95-1.01)	0.993
Beta-blockers						
Monday	1.1 (1.07-1.12)	<0.001	1.1 (1.07-1.13)	<0.001	1.09 (1.04-1.13)	0.107
Tuesday	1.04 (1.02-1.06)	0.237	1.04 (1.01-1.07)	0.603	1.03 (0.99-1.08)	0.993
Wednesday	1.05 (1.03-1.07)	0.007	1.05 (1.02-1.08)	0.035	1.04 (1-1.09)	0.993
Thursday	1.06 (1.03-1.08)	<0.001	1.06 (1.04-1.09)	<0.001	1.04 (0.99-1.08)	0.993
Friday	1.05 (1.03-1.07)	0.009	1.05 (1.02-1.08)	0.035	1.04 (0.99-1.08)	0.993
Saturday	0.99 (0.97-1.02)	1.000	0.99 (0.97-1.02)	0.998	1 (0.96-1.05)	0.993
No Beta-blockers						
Monday	1.09 (1.07-1.11)	<0.001	1.13 (1.1-1.16)	<0.001	1.04 (1.01-1.07)	0.993
Tuesday	1.01 (0.99-1.02)	1.000	1.03 (1-1.05)	0.998	0.98 (0.95-1)	0.993
Wednesday	1.01 (0.99-1.02)	1.000	1.03 (1.01-1.06)	0.998	0.97 (0.94-1)	0.993
Thursday	1.01 (0.99-1.03)	1.000	1.03 (1.01-1.06)	0.998	0.98 (0.95-1)	0.993
Friday	1 (0.98-1.02)	1.000	1.02 (1-1.04)	0.998	0.97 (0.95-1)	0.993
Saturday	0.96 (0.95-0.98)	0.056	0.96 (0.93-0.98)	0.149	0.98 (0.95-1)	0.993
Ca-inhibitors						
Monday	1.06 (1.03-1.1)	0.058	1.07 (1.03-1.11)	0.218	1.06 (0.99-1.12)	0.993
Tuesday	1.01 (0.97-1.04)	1.000	1.01 (0.97-1.05)	0.998	1 (0.94-1.07)	0.993
Wednesday	1.02 (0.98-1.05)	1.000	1.02 (0.98-1.06)	0.998	1.02 (0.96-1.09)	0.993
Thursday	1.04 (1-1.07)	1.000	1.05 (1.01-1.09)	0.998	1.01 (0.95-1.07)	0.993
Friday	1.04 (1-1.07)	1.000	1.04 (1-1.08)	0.998	1.02 (0.96-1.09)	0.993
Saturday	0.98 (0.95-1.01)	1.000	0.98 (0.95-1.02)	0.998	0.96 (0.9-1.03)	0.993
No Ca-inhibitors						
Monday	1.1 (1.08-1.12)	<0.001	1.13 (1.11-1.15)	<0.001	1.05 (1.02-1.08)	0.104
Tuesday	1.02 (1.01-1.04)	1.000	1.04 (1.02-1.06)	0.027	0.99 (0.97-1.02)	0.993
Wednesday	1.03 (1.01-1.04)	0.510	1.05 (1.03-1.07)	0.001	0.99 (0.96-1.01)	0.993
Thursday	1.03 (1.01-1.04)	0.200	1.05 (1.03-1.07)	0.001	0.99 (0.97-1.02)	0.993
Friday	1.02 (1-1.03)	1.000	1.03 (1.01-1.05)	0.001	0.99 (0.96-1.01)	0.993
Saturday	0.98 (0.96-0.99)	0.834	0.97 (0.95-0.99)	0.807	0.99 (0.96-1.01)	0.993
Aspirin						
Monday	1.09 (1.07-1.11)	<0.001	1.09 (1.07-1.12)	<0.001	1.07 (1.03-1.12)	0.709
Tuesday	1.04 (1.01-1.06)	0.401	1.04 (1.02-1.07)	0.483	1.02 (0.98-1.07)	0.993

Wednesday		1.04 (1.02-1.06)	0.045	1.05 (1.02-1.07)	0.044	1.02 (0.98-1.07)	0.993
Thursday		1.06 (1.04-1.08)	<0.001	1.06 (1.04-1.09)	0.001	1.06 (1.01-1.1)	0.993
Friday		1.05 (1.03-1.07)	0.002	1.05 (1.03-1.08)	0.044	1.04 (0.99-1.08)	0.993
Saturday		0.98 (0.96-1.01)	1.000	0.98 (0.96-1.01)	0.998	0.99 (0.94-1.03)	0.993
No Aspirin							
Monday		1.1 (1.08-1.12)	<0.001	1.14 (1.11-1.16)	<0.001	1.04 (1.01-1.07)	0.993
Tuesday		1.01 (0.99-1.03)	1.000	1.03 (1-1.05)	0.998	0.98 (0.95-1.01)	0.993
Wednesday		1.01 (0.99-1.03)	1.000	1.03 (1.01-1.06)	0.998	0.98 (0.95-1)	0.993
Thursday		1.01 (0.99-1.02)	1.000	1.03 (1.01-1.06)	0.998	0.97 (0.94-0.99)	0.993
Friday		1 (0.98-1.02)	1.000	1.01 (0.99-1.04)	0.998	0.97 (0.95-1)	0.993
Saturday		0.97 (0.95-0.99)	0.670	0.96 (0.94-0.99)	0.718	0.98 (0.96-1.01)	0.993
ACE-I/ARB							
Monday		1.1 (1.07-1.12)	<0.001	1.1 (1.07-1.13)	<0.001	1.07 (1.02-1.13)	0.993
Tuesday		1.04 (1.02-1.07)	0.505	1.03 (1-1.06)	0.998	1.07 (1.02-1.12)	0.993
Wednesday		1.04 (1.01-1.06)	1.000	1.04 (1.01-1.07)	0.998	1.03 (0.98-1.08)	0.993
Thursday		1.04 (1.02-1.07)	0.327	1.05 (1.02-1.08)	0.152	1.01 (0.96-1.07)	0.993
Friday		1.03 (1-1.06)	1.000	1.03 (1.01-1.06)	0.998	1.01 (0.96-1.07)	0.993
Saturday		0.98 (0.96-1.01)	1.000	0.98 (0.96-1.01)	0.998	0.98 (0.93-1.03)	0.993
No ACE-I/ARB							
Monday		1.09 (1.07-1.11)	<0.001	1.12 (1.1-1.15)	<0.001	1.05 (1.02-1.07)	0.756
Tuesday		1.01 (0.99-1.03)	1.000	1.03 (1.01-1.06)	0.814	0.97 (0.95-1)	0.993
Wednesday		1.02 (1-1.04)	1.000	1.04 (1.02-1.07)	0.040	0.98 (0.95-1.01)	0.993
Thursday		1.02 (1.01-1.04)	1.000	1.05 (1.02-1.07)	0.018	0.99 (0.96-1.02)	0.993
Friday		1.02 (1-1.03)	1.000	1.03 (1.01-1.06)	0.040	0.99 (0.96-1.01)	0.993
Saturday		0.97 (0.96-0.99)	0.805	0.97 (0.95-0.99)	0.998	0.98 (0.96-1.01)	0.993
Statins							
Monday		1.09 (1.06-1.12)	<0.001	1.11 (1.07-1.14)	<0.001	1.05 (0.99-1.11)	0.993
Tuesday		1.05 (1.02-1.08)	0.153	1.05 (1.02-1.08)	0.398	1.04 (0.99-1.1)	0.993
Wednesday		1.04 (1.02-1.07)	0.548	1.05 (1.02-1.08)	0.938	1.03 (0.98-1.09)	0.993
Thursday		1.05 (1.03-1.08)	0.051	1.06 (1.03-1.1)	0.042	1.03 (0.97-1.09)	0.993
Friday		1.05 (1.02-1.08)	0.099	1.06 (1.03-1.1)	0.938	1.01 (0.95-1.07)	0.993
Saturday		0.98 (0.95-1)	1.000	0.97 (0.94-1)	0.998	0.99 (0.94-1.05)	0.993
No Statins							
Monday		1.09 (1.08-1.11)	<0.001	1.12 (1.1-1.14)	<0.001	1.05 (1.02-1.08)	0.100
Tuesday		1.01 (0.99-1.02)	1.000	1.02 (1-1.05)	0.998	0.98 (0.96-1.01)	0.993
Wednesday		1.02 (1-1.03)	1.000	1.04 (1.02-1.06)	0.111	0.98 (0.96-1.01)	0.993
Thursday		1.02 (1-1.04)	1.000	1.04 (1.02-1.06)	0.041	0.99 (0.96-1.01)	0.993
Friday		1.01 (0.99-1.03)	1.000	1.02 (1-1.04)	0.111	0.99 (0.96-1.02)	0.993
Saturday		0.98 (0.96-0.99)	1.000	0.97 (0.95-0.99)	0.998	0.98 (0.96-1.01)	0.993
Men		1		1		1	
	01:00	0.89 (0.86-0.93)	<0.001	0.88 (0.84-0.92)	<0.001	1.01 (0.95-1.08)	0.993
	02:00	0.94 (0.91-0.98)	0.406	0.92 (0.88-0.96)	0.030	1.07 (1-1.14)	0.993
	03:00	0.93 (0.9-0.97)	0.042	0.9 (0.86-0.94)	0.001	1.05 (0.99-1.12)	0.993
	04:00	0.88 (0.85-0.91)	<0.001	0.86 (0.82-0.89)	<0.001	1.04 (0.98-1.11)	0.993
	05:00	0.85 (0.82-0.88)	<0.001	0.82 (0.78-0.86)	<0.001	1.04 (0.98-1.11)	0.993
	06:00	0.96 (0.92-0.99)	1.000	0.9 (0.86-0.94)	0.001	1.06 (1-1.13)	0.993
	07:00	1.12 (1.09-1.16)	<0.001	1.03 (0.99-1.07)	0.998	1.15 (1.08-1.22)	0.004
	08:00	1.63 (1.58-1.68)	<0.001	1.45 (1.4-1.5)	<0.001	1.33 (1.26-1.4)	<0.001

	09:00	1.24 (1.2-1.28)	<0.001	1.1 (1.05-1.14)	0.003	1.26 (1.19-1.33)	<0.001
	10:00	1.33 (1.28-1.37)	<0.001	1.14 (1.1-1.19)	<0.001	1.32 (1.25-1.39)	<0.001
	11:00	1.08 (1.05-1.12)	0.001	0.96 (0.92-1)	0.998	1.25 (1.18-1.33)	<0.001
	12:00	1.28 (1.24-1.32)	<0.001	1.15 (1.1-1.19)	<0.001	1.25 (1.18-1.32)	<0.001
	13:00	0.97 (0.94-1.01)	1.000	0.88 (0.84-0.92)	<0.001	1.17 (1.1-1.24)	<0.001
	14:00	1.01 (0.98-1.05)	1.000	0.92 (0.88-0.96)	0.035	1.16 (1.1-1.24)	<0.001
	15:00	1.02 (0.98-1.05)	1.000	0.94 (0.9-0.98)	0.998	1.12 (1.05-1.18)	0.238
	16:00	0.93 (0.9-0.96)	0.016	0.87 (0.83-0.91)	<0.001	1.1 (1.04-1.17)	0.993
	17:00	0.86 (0.83-0.89)	<0.001	0.81 (0.77-0.85)	<0.001	1.06 (1-1.13)	0.993
	18:00	0.88 (0.85-0.92)	<0.001	0.85 (0.81-0.89)	<0.001	1.03 (0.97-1.1)	0.993
	19:00	0.85 (0.82-0.88)	<0.001	0.82 (0.78-0.86)	<0.001	1.02 (0.95-1.08)	0.993
	20:00	0.94 (0.91-0.98)	0.433	0.92 (0.88-0.96)	0.023	1 (0.94-1.07)	0.993
	21:00	0.87 (0.84-0.91)	<0.001	0.85 (0.81-0.88)	<0.001	1.01 (0.94-1.07)	0.993
	22:00	0.93 (0.89-0.96)	0.006	0.9 (0.86-0.94)	<0.001	1.01 (0.95-1.07)	0.993
	23:00	0.96 (0.92-0.99)	1.000	0.93 (0.89-0.97)	0.113	1.07 (1.01-1.14)	0.993
Women		1		1		1	
	01:00	0.87 (0.83-0.91)	<0.001	0.88 (0.83-0.93)	0.004	0.93 (0.85-1.02)	0.993
	02:00	0.87 (0.83-0.91)	<0.001	0.88 (0.83-0.93)	0.001	0.92 (0.84-1)	0.993
	03:00	0.87 (0.83-0.91)	<0.001	0.86 (0.82-0.91)	<0.001	0.95 (0.87-1.03)	0.993
	04:00	0.83 (0.79-0.87)	<0.001	0.85 (0.8-0.9)	<0.001	0.91 (0.83-0.99)	0.993
	05:00	0.79 (0.75-0.83)	<0.001	0.8 (0.76-0.85)	<0.001	0.9 (0.82-0.98)	0.993
	06:00	0.93 (0.89-0.98)	0.820	0.9 (0.86-0.95)	0.107	1 (0.92-1.09)	0.993
	07:00	1.05 (1-1.09)	1.000	0.98 (0.93-1.04)	0.998	1.03 (0.96-1.12)	0.993
	08:00	1.51 (1.45-1.57)	<0.001	1.37 (1.31-1.43)	<0.001	1.19 (1.1-1.28)	0.004
	09:00	1.05 (1.01-1.1)	1.000	0.95 (0.9-1)	0.998	1.06 (0.98-1.14)	0.993
	10:00	1.1 (1.05-1.14)	0.010	0.97 (0.93-1.03)	0.998	1.06 (0.98-1.15)	0.993
	11:00	0.85 (0.82-0.89)	<0.001	0.79 (0.74-0.83)	<0.001	0.96 (0.89-1.04)	0.993
	12:00	1.14 (1.09-1.19)	<0.001	1.06 (1.01-1.12)	0.998	1.06 (0.98-1.14)	0.993
	13:00	0.81 (0.77-0.85)	<0.001	0.8 (0.75-0.84)	<0.001	0.88 (0.81-0.95)	0.993
	14:00	0.87 (0.83-0.91)	<0.001	0.84 (0.79-0.89)	<0.001	0.93 (0.85-1.01)	0.993
	15:00	0.97 (0.93-1.02)	1.000	0.93 (0.88-0.98)	0.998	1.03 (0.95-1.11)	0.993
	16:00	0.86 (0.83-0.91)	<0.001	0.86 (0.81-0.91)	<0.001	0.95 (0.87-1.03)	0.993
	17:00	0.83 (0.79-0.87)	<0.001	0.86 (0.81-0.91)	<0.001	0.9 (0.82-0.98)	0.993
	18:00	0.9 (0.86-0.94)	0.003	0.9 (0.85-0.95)	0.044	1 (0.92-1.09)	0.993
	19:00	0.87 (0.83-0.91)	<0.001	0.88 (0.83-0.93)	0.002	0.97 (0.89-1.06)	0.993
	20:00	1.03 (0.99-1.08)	1.000	1.03 (0.98-1.08)	0.998	1.04 (0.95-1.13)	0.993
	21:00	0.91 (0.87-0.96)	0.050	0.93 (0.88-0.98)	0.998	0.96 (0.88-1.05)	0.993
	22:00	0.98 (0.94-1.03)	1.000	0.98 (0.93-1.03)	0.998	1.01 (0.93-1.1)	0.993
	23:00	0.89 (0.85-0.94)	0.001	0.91 (0.86-0.96)	0.130	0.91 (0.83-0.99)	0.993
Age≥75yo		1		1		1	
	01:00	0.87 (0.83-0.9)	<0.001	0.88 (0.84-0.93)	<0.001	0.93 (0.86-1.01)	0.993
	02:00	0.9 (0.87-0.94)	<0.001	0.91 (0.87-0.96)	0.044	0.96 (0.89-1.05)	0.993
	03:00	0.89 (0.86-0.93)	<0.001	0.9 (0.86-0.94)	0.003	0.94 (0.87-1.03)	0.993
	04:00	0.83 (0.79-0.86)	<0.001	0.85 (0.81-0.89)	<0.001	0.91 (0.84-0.99)	0.993
	05:00	0.8 (0.77-0.84)	<0.001	0.81 (0.78-0.86)	<0.001	0.95 (0.88-1.03)	0.993
	06:00	0.91 (0.88-0.95)	0.004	0.9 (0.86-0.95)	0.005	0.98 (0.91-1.06)	0.993
	07:00	1.02 (0.98-1.06)	1.000	0.97 (0.92-1.01)	0.998	1.04 (0.96-1.12)	0.993
	08:00	1.53 (1.48-1.58)	<0.001	1.35 (1.3-1.41)	<0.001	1.34 (1.25-1.43)	<0.001
	09:00	1.01 (0.97-1.04)	1.000	0.9 (0.86-0.94)	0.002	1.1 (1.02-1.19)	0.993

	10:00	1.05 (1.02-1.09)	1.000	0.93 (0.89-0.97)	0.423	1.11 (1.04-1.2)	0.993
	11:00	0.81 (0.78-0.84)	<0.001	0.74 (0.7-0.77)	<0.001	1.03 (0.95-1.11)	0.993
	12:00	1.06 (1.02-1.1)	0.505	0.97 (0.93-1.01)	0.998	1.09 (1.02-1.18)	0.993
	13:00	0.75 (0.72-0.78)	<0.001	0.72 (0.69-0.76)	<0.001	0.9 (0.83-0.98)	0.993
	14:00	0.79 (0.75-0.82)	<0.001	0.75 (0.71-0.78)	<0.001	0.92 (0.85-0.99)	0.993
	15:00	0.85 (0.82-0.88)	<0.001	0.81 (0.77-0.84)	<0.001	0.98 (0.91-1.06)	0.993
	16:00	0.77 (0.74-0.81)	<0.001	0.75 (0.72-0.79)	<0.001	0.94 (0.87-1.02)	0.993
	17:00	0.73 (0.7-0.77)	<0.001	0.74 (0.71-0.78)	<0.001	0.87 (0.8-0.95)	0.897
	18:00	0.81 (0.78-0.84)	<0.001	0.81 (0.77-0.85)	<0.001	0.94 (0.87-1.02)	0.993
	19:00	0.76 (0.73-0.79)	<0.001	0.76 (0.72-0.8)	<0.001	0.91 (0.83-0.99)	0.993
	20:00	0.93 (0.89-0.96)	0.054	0.93 (0.89-0.97)	0.252	0.95 (0.88-1.03)	0.993
	21:00	0.87 (0.84-0.91)	<0.001	0.88 (0.84-0.92)	<0.001	0.95 (0.87-1.03)	0.993
	22:00	0.92 (0.88-0.95)	0.004	0.91 (0.87-0.95)	0.018	0.97 (0.89-1.05)	0.993
	23:00	0.89 (0.85-0.92)	<0.001	0.92 (0.88-0.96)	0.060	0.86 (0.79-0.94)	0.431
Age<75yo		1		1		1	
	01:00	0.9 (0.87-0.94)	<0.001	0.88 (0.83-0.92)	<0.001	1.02 (0.95-1.09)	0.993
	02:00	0.93 (0.89-0.97)	0.096	0.89 (0.85-0.93)	0.001	1.05 (0.98-1.12)	0.993
	03:00	0.93 (0.89-0.96)	0.081	0.87 (0.83-0.91)	<0.001	1.06 (1-1.14)	0.993
	04:00	0.89 (0.86-0.93)	<0.001	0.86 (0.81-0.9)	<0.001	1.05 (0.98-1.12)	0.993
	05:00	0.85 (0.82-0.89)	<0.001	0.81 (0.76-0.85)	<0.001	1.02 (0.95-1.09)	0.993
	06:00	0.98 (0.94-1.02)	1.000	0.9 (0.86-0.95)	0.013	1.08 (1.01-1.15)	0.993
	07:00	1.17 (1.13-1.21)	<0.001	1.07 (1.02-1.12)	0.969	1.15 (1.08-1.22)	0.005
	08:00	1.64 (1.58-1.69)	<0.001	1.5 (1.44-1.56)	<0.001	1.24 (1.17-1.31)	<0.001
	09:00	1.34 (1.29-1.39)	<0.001	1.21 (1.15-1.26)	<0.001	1.24 (1.17-1.31)	<0.001
	10:00	1.43 (1.38-1.48)	<0.001	1.25 (1.2-1.31)	<0.001	1.3 (1.23-1.38)	<0.001
	11:00	1.19 (1.14-1.23)	<0.001	1.08 (1.03-1.13)	0.714	1.23 (1.16-1.3)	<0.001
	12:00	1.39 (1.35-1.44)	<0.001	1.29 (1.23-1.35)	<0.001	1.24 (1.17-1.31)	<0.001
	13:00	1.08 (1.04-1.12)	0.033	0.99 (0.94-1.04)	0.998	1.17 (1.1-1.25)	<0.001
	14:00	1.14 (1.1-1.18)	<0.001	1.06 (1.01-1.11)	0.998	1.19 (1.12-1.26)	<0.001
	15:00	1.15 (1.11-1.19)	<0.001	1.09 (1.04-1.15)	0.071	1.15 (1.09-1.23)	0.003
	16:00	1.04 (1-1.08)	1.000	1 (0.95-1.05)	0.998	1.12 (1.05-1.19)	0.434
	17:00	0.96 (0.93-1)	1.000	0.93 (0.88-0.97)	0.998	1.09 (1.02-1.16)	0.993
	18:00	0.97 (0.93-1.01)	1.000	0.94 (0.89-0.99)	0.998	1.07 (1.01-1.14)	0.993
	19:00	0.96 (0.92-0.99)	1.000	0.94 (0.89-0.99)	0.998	1.06 (1-1.13)	0.993
	20:00	1.03 (0.99-1.07)	1.000	1 (0.96-1.05)	0.998	1.06 (0.99-1.13)	0.993
	21:00	0.91 (0.87-0.95)	0.002	0.88 (0.84-0.92)	<0.001	1.02 (0.95-1.09)	0.993
	22:00	0.98 (0.94-1.02)	1.000	0.95 (0.91-1)	0.998	1.04 (0.97-1.11)	0.993
	23:00	0.98 (0.94-1.02)	1.000	0.92 (0.88-0.97)	0.323	1.11 (1.04-1.19)	0.718
Current smoker		1		1		1	
	01:00	0.92 (0.87-0.98)	1.000	0.88 (0.81-0.96)	0.998	1.04 (0.94-1.15)	0.993
	02:00	0.96 (0.91-1.03)	1.000	0.92 (0.84-0.99)	0.998	1.07 (0.97-1.17)	0.993
	03:00	0.91 (0.86-0.97)	1.000	0.88 (0.81-0.96)	0.998	0.97 (0.88-1.08)	0.993
	04:00	0.86 (0.8-0.91)	0.002	0.82 (0.75-0.89)	0.002	0.97 (0.88-1.08)	0.993
	05:00	0.86 (0.8-0.92)	0.002	0.81 (0.74-0.88)	0.001	0.98 (0.88-1.08)	0.993
	06:00	0.92 (0.86-0.98)	1.000	0.82 (0.75-0.89)	0.001	1.01 (0.92-1.11)	0.993
	07:00	1.05 (0.99-1.12)	1.000	0.98 (0.9-1.06)	0.998	0.98 (0.89-1.08)	0.993
	08:00	1.44 (1.36-1.52)	<0.001	1.31 (1.22-1.4)	<0.001	1.09 (1-1.19)	0.993
	09:00	1.14 (1.08-1.21)	0.006	1.01 (0.94-1.09)	0.998	1.04 (0.95-1.13)	0.993
	10:00	1.25 (1.18-1.32)	<0.001	1.11 (1.03-1.2)	0.998	1.07 (0.98-1.17)	0.993

	11:00	1.07 (1-1.13)	1.000	0.94 (0.86-1.02)	0.998	1.07 (0.98-1.17)	0.993
	12:00	1.3 (1.22-1.37)	<0.001	1.19 (1.1-1.28)	0.004	1.13 (1.04-1.24)	0.993
	13:00	1.03 (0.97-1.09)	1.000	0.9 (0.83-0.98)	0.998	1.1 (1-1.21)	0.993
	14:00	1.08 (1.02-1.15)	1.000	0.96 (0.89-1.05)	0.998	1.12 (1.02-1.23)	0.993
	15:00	1.1 (1.03-1.16)	0.864	0.97 (0.89-1.05)	0.998	1.16 (1.05-1.27)	0.993
	16:00	0.96 (0.9-1.02)	1.000	0.88 (0.81-0.96)	0.998	1.05 (0.95-1.15)	0.993
	17:00	0.95 (0.89-1.01)	1.000	0.91 (0.83-0.99)	0.998	1.03 (0.93-1.13)	0.993
	18:00	0.95 (0.89-1.01)	1.000	0.9 (0.83-0.98)	0.998	1.05 (0.95-1.15)	0.993
	19:00	0.91 (0.85-0.97)	0.874	0.88 (0.81-0.96)	0.998	0.99 (0.9-1.09)	0.993
	20:00	1.04 (0.98-1.1)	1.000	1.01 (0.93-1.09)	0.998	1.06 (0.96-1.17)	0.993
	21:00	0.93 (0.88-1)	1.000	0.89 (0.82-0.97)	0.998	1.04 (0.94-1.14)	0.993
	22:00	0.98 (0.92-1.04)	1.000	0.95 (0.88-1.03)	0.998	1.03 (0.93-1.13)	0.993
	23:00	0.99 (0.93-1.06)	1.000	0.91 (0.84-0.99)	0.998	1.13 (1.02-1.24)	0.993
Non-smoker		1		1		1	
	01:00	0.91 (0.88-0.94)	<0.001	0.91 (0.88-0.95)	0.005	0.99 (0.93-1.06)	0.993
	02:00	0.93 (0.9-0.96)	0.016	0.93 (0.89-0.97)	0.082	1.02 (0.96-1.09)	0.993
	03:00	0.94 (0.91-0.97)	0.101	0.92 (0.88-0.95)	0.007	1.06 (1-1.13)	0.993
	04:00	0.89 (0.86-0.92)	<0.001	0.89 (0.85-0.92)	<0.001	1.04 (0.98-1.11)	0.993
	05:00	0.86 (0.83-0.89)	<0.001	0.84 (0.81-0.88)	<0.001	1.04 (0.97-1.1)	0.993
	06:00	0.99 (0.96-1.03)	1.000	0.96 (0.92-1)	0.998	1.09 (1.02-1.16)	0.993
	07:00	1.15 (1.12-1.19)	<0.001	1.06 (1.02-1.1)	0.434	1.21 (1.14-1.28)	<0.001
	08:00	1.67 (1.63-1.72)	<0.001	1.49 (1.45-1.54)	<0.001	1.38 (1.3-1.45)	<0.001
	09:00	1.23 (1.2-1.27)	<0.001	1.1 (1.06-1.14)	0.001	1.29 (1.22-1.37)	<0.001
	10:00	1.29 (1.25-1.33)	<0.001	1.12 (1.08-1.16)	<0.001	1.32 (1.25-1.39)	<0.001
	11:00	1.02 (0.99-1.05)	1.000	0.92 (0.88-0.95)	0.006	1.22 (1.15-1.29)	<0.001
	12:00	1.24 (1.21-1.28)	<0.001	1.13 (1.09-1.18)	<0.001	1.22 (1.15-1.29)	<0.001
	13:00	0.93 (0.9-0.96)	0.007	0.88 (0.84-0.92)	<0.001	1.09 (1.03-1.16)	0.993
	14:00	0.97 (0.94-1.01)	1.000	0.91 (0.88-0.95)	0.002	1.11 (1.04-1.17)	0.519
	15:00	1.01 (0.98-1.04)	1.000	0.96 (0.92-1)	0.998	1.1 (1.03-1.16)	0.993
	16:00	0.93 (0.9-0.96)	0.006	0.9 (0.87-0.94)	<0.001	1.08 (1.01-1.14)	0.993
	17:00	0.85 (0.82-0.88)	<0.001	0.84 (0.8-0.87)	<0.001	1.01 (0.95-1.08)	0.993
	18:00	0.9 (0.87-0.93)	<0.001	0.89 (0.85-0.92)	<0.001	1.04 (0.97-1.1)	0.993
	19:00	0.87 (0.84-0.9)	<0.001	0.86 (0.83-0.9)	<0.001	1.03 (0.97-1.1)	0.993
	20:00	0.99 (0.96-1.02)	1.000	0.98 (0.95-1.02)	0.998	1.02 (0.95-1.08)	0.993
	21:00	0.91 (0.88-0.94)	<0.001	0.9 (0.87-0.94)	<0.001	1 (0.94-1.07)	0.993
	22:00	0.97 (0.94-1.01)	1.000	0.96 (0.92-0.99)	0.998	1.04 (0.98-1.11)	0.993
	23:00	0.95 (0.92-0.98)	0.662	0.95 (0.91-0.99)	0.998	1 (0.94-1.07)	0.993
Diabetes		1		1		1	
	01:00	0.84 (0.8-0.89)	<0.001	0.85 (0.8-0.91)	0.002	0.92 (0.81-1.04)	0.993
	02:00	0.86 (0.82-0.91)	<0.001	0.89 (0.83-0.95)	0.119	0.88 (0.78-1)	0.993
	03:00	0.82 (0.77-0.87)	<0.001	0.82 (0.77-0.88)	<0.001	0.89 (0.79-1)	0.993
	04:00	0.78 (0.74-0.83)	<0.001	0.79 (0.74-0.85)	<0.001	0.92 (0.81-1.04)	0.993
	05:00	0.74 (0.69-0.79)	<0.001	0.77 (0.71-0.82)	<0.001	0.84 (0.74-0.95)	0.993
	06:00	0.84 (0.8-0.89)	<0.001	0.83 (0.78-0.89)	<0.001	0.91 (0.81-1.02)	0.993
	07:00	0.95 (0.9-1)	1.000	0.9 (0.84-0.96)	0.297	0.98 (0.87-1.09)	0.993
	08:00	1.41 (1.34-1.48)	<0.001	1.3 (1.23-1.37)	<0.001	1.1 (0.99-1.22)	0.993
	09:00	0.97 (0.92-1.02)	1.000	0.91 (0.85-0.97)	0.695	0.98 (0.88-1.09)	0.993
	10:00	1.01 (0.95-1.06)	1.000	0.9 (0.85-0.96)	0.387	1.04 (0.94-1.16)	0.993
	11:00	0.74 (0.7-0.78)	<0.001	0.7 (0.65-0.75)	<0.001	0.88 (0.79-0.99)	0.993

	12:00	1.01 (0.96-1.06)	1.000	0.96 (0.9-1.02)	0.998	0.95 (0.85-1.06)	0.993
	13:00	0.71 (0.67-0.76)	<0.001	0.69 (0.64-0.74)	<0.001	0.86 (0.76-0.96)	0.993
	14:00	0.77 (0.73-0.82)	<0.001	0.77 (0.72-0.82)	<0.001	0.82 (0.73-0.93)	0.761
	15:00	0.81 (0.77-0.86)	<0.001	0.8 (0.75-0.86)	<0.001	0.87 (0.77-0.98)	0.993
	16:00	0.73 (0.68-0.77)	<0.001	0.75 (0.7-0.81)	<0.001	0.76 (0.67-0.86)	0.010
	17:00	0.73 (0.68-0.77)	<0.001	0.74 (0.69-0.8)	<0.001	0.85 (0.76-0.96)	0.993
	18:00	0.8 (0.75-0.85)	<0.001	0.8 (0.75-0.86)	<0.001	0.92 (0.82-1.04)	0.993
	19:00	0.77 (0.72-0.81)	<0.001	0.77 (0.72-0.83)	<0.001	0.9 (0.8-1.01)	0.993
	20:00	0.96 (0.91-1.01)	1.000	0.97 (0.91-1.03)	0.998	0.95 (0.85-1.07)	0.993
	21:00	0.86 (0.81-0.91)	<0.001	0.87 (0.82-0.93)	0.016	0.91 (0.8-1.02)	0.993
	22:00	0.9 (0.85-0.96)	0.158	0.92 (0.86-0.98)	0.998	0.89 (0.79-1)	0.993
	23:00	0.89 (0.84-0.94)	0.032	0.91 (0.86-0.97)	0.998	0.89 (0.79-1)	0.993
No Diabetes		1		1		1	
	01:00	0.9 (0.87-0.93)	<0.001	0.89 (0.86-0.93)	<0.001	1 (0.94-1.06)	0.993
	02:00	0.93 (0.91-0.97)	0.017	0.91 (0.88-0.95)	0.002	1.04 (0.99-1.1)	0.993
	03:00	0.94 (0.91-0.97)	0.061	0.91 (0.88-0.95)	0.002	1.04 (0.99-1.1)	0.993
	04:00	0.89 (0.86-0.92)	<0.001	0.88 (0.84-0.91)	<0.001	1.01 (0.96-1.07)	0.993
	05:00	0.86 (0.83-0.89)	<0.001	0.83 (0.8-0.87)	<0.001	1.02 (0.97-1.08)	0.993
	06:00	0.98 (0.95-1.02)	1.000	0.93 (0.9-0.97)	0.137	1.07 (1.01-1.13)	0.993
	07:00	1.15 (1.11-1.18)	<0.001	1.06 (1.02-1.1)	0.527	1.14 (1.08-1.2)	0.001
	08:00	1.65 (1.6-1.69)	<0.001	1.47 (1.42-1.52)	<0.001	1.32 (1.26-1.39)	<0.001
	09:00	1.24 (1.2-1.28)	<0.001	1.09 (1.05-1.13)	0.001	1.23 (1.17-1.3)	<0.001
	10:00	1.32 (1.28-1.36)	<0.001	1.15 (1.11-1.19)	<0.001	1.27 (1.21-1.33)	<0.001
	11:00	1.08 (1.05-1.12)	<0.001	0.97 (0.93-1)	0.998	1.21 (1.15-1.27)	<0.001
	12:00	1.3 (1.26-1.34)	<0.001	1.18 (1.14-1.22)	<0.001	1.23 (1.17-1.3)	<0.001
	13:00	0.98 (0.95-1.01)	1.000	0.9 (0.87-0.94)	<0.001	1.11 (1.06-1.18)	0.050
	14:00	1.03 (0.99-1.06)	1.000	0.94 (0.9-0.97)	0.373	1.14 (1.08-1.2)	0.001
	15:00	1.06 (1.03-1.1)	0.050	0.99 (0.96-1.03)	0.998	1.13 (1.07-1.2)	0.003
	16:00	0.97 (0.93-1)	1.000	0.91 (0.88-0.95)	0.002	1.11 (1.05-1.18)	0.088
	17:00	0.89 (0.86-0.92)	<0.001	0.86 (0.82-0.89)	<0.001	1.04 (0.98-1.09)	0.993
	18:00	0.92 (0.89-0.95)	<0.001	0.9 (0.86-0.93)	<0.001	1.04 (0.99-1.1)	0.993
	19:00	0.89 (0.86-0.92)	<0.001	0.87 (0.83-0.9)	<0.001	1.02 (0.96-1.08)	0.993
	20:00	0.99 (0.96-1.02)	1.000	0.96 (0.93-1)	0.998	1.03 (0.97-1.09)	0.993
	21:00	0.9 (0.87-0.93)	<0.001	0.88 (0.85-0.92)	<0.001	1.01 (0.95-1.07)	0.993
	22:00	0.96 (0.93-0.99)	1.000	0.93 (0.9-0.97)	0.157	1.04 (0.98-1.1)	0.993
	23:00	0.95 (0.92-0.98)	0.389	0.92 (0.89-0.96)	0.018	1.04 (0.98-1.1)	0.993
Hypertension		1		1		1	
	01:00	0.88 (0.84-0.91)	<0.001	0.9 (0.86-0.94)	0.006	0.91 (0.84-0.99)	0.993
	02:00	0.89 (0.86-0.93)	<0.001	0.9 (0.86-0.95)	0.011	0.95 (0.88-1.03)	0.993
	03:00	0.89 (0.85-0.93)	<0.001	0.88 (0.84-0.92)	<0.001	0.98 (0.9-1.06)	0.993
	04:00	0.82 (0.78-0.85)	<0.001	0.82 (0.78-0.86)	<0.001	0.95 (0.87-1.03)	0.993
	05:00	0.78 (0.75-0.82)	<0.001	0.76 (0.73-0.81)	<0.001	0.98 (0.9-1.06)	0.993
	06:00	0.91 (0.87-0.95)	0.002	0.89 (0.84-0.93)	<0.001	0.98 (0.91-1.06)	0.993
	07:00	1 (0.97-1.04)	1.000	0.94 (0.9-0.99)	0.998	1.02 (0.95-1.1)	0.993
	08:00	1.49 (1.44-1.55)	<0.001	1.36 (1.31-1.42)	<0.001	1.18 (1.1-1.26)	0.003
	09:00	1.05 (1.01-1.09)	1.000	0.96 (0.92-1.01)	0.998	1.06 (0.99-1.14)	0.993
	10:00	1.1 (1.06-1.15)	<0.001	0.98 (0.94-1.02)	0.998	1.11 (1.03-1.19)	0.993
	11:00	0.85 (0.82-0.88)	<0.001	0.79 (0.75-0.83)	<0.001	0.98 (0.91-1.06)	0.993
	12:00	1.1 (1.06-1.15)	<0.001	1.02 (0.97-1.06)	0.998	1.08 (1-1.16)	0.993

	13:00	0.79 (0.75-0.82)	<0.001	0.76 (0.72-0.8)	<0.001	0.92 (0.85-0.99)	0.993
	14:00	0.86 (0.82-0.89)	<0.001	0.81 (0.77-0.85)	<0.001	0.98 (0.91-1.06)	0.993
	15:00	0.89 (0.85-0.93)	<0.001	0.86 (0.82-0.9)	<0.001	0.95 (0.88-1.02)	0.993
	16:00	0.79 (0.76-0.83)	<0.001	0.78 (0.74-0.82)	<0.001	0.9 (0.83-0.97)	0.993
	17:00	0.78 (0.74-0.81)	<0.001	0.77 (0.73-0.81)	<0.001	0.92 (0.85-1)	0.993
	18:00	0.83 (0.79-0.86)	<0.001	0.82 (0.78-0.86)	<0.001	0.95 (0.88-1.03)	0.993
	19:00	0.81 (0.78-0.85)	<0.001	0.81 (0.77-0.85)	<0.001	0.95 (0.87-1.03)	0.993
	20:00	0.93 (0.89-0.96)	0.066	0.92 (0.87-0.96)	0.088	0.96 (0.89-1.04)	0.993
	21:00	0.87 (0.83-0.9)	<0.001	0.86 (0.82-0.9)	<0.001	0.97 (0.89-1.05)	0.993
	22:00	0.93 (0.9-0.97)	0.261	0.93 (0.88-0.97)	0.351	0.99 (0.91-1.07)	0.993
	23:00	0.91 (0.87-0.95)	0.003	0.9 (0.86-0.95)	0.012	0.98 (0.91-1.07)	0.993
No Hypertension		1		1		1	
	01:00	0.9 (0.86-0.94)	<0.001	0.87 (0.83-0.91)	<0.001	1.04 (0.97-1.11)	0.993
	02:00	0.94 (0.91-0.98)	0.805	0.91 (0.87-0.96)	0.049	1.06 (0.99-1.13)	0.993
	03:00	0.93 (0.9-0.97)	0.161	0.9 (0.85-0.94)	0.004	1.04 (0.97-1.12)	0.993
	04:00	0.91 (0.87-0.94)	<0.001	0.89 (0.85-0.94)	0.003	1.03 (0.96-1.1)	0.993
	05:00	0.88 (0.84-0.92)	<0.001	0.87 (0.82-0.91)	<0.001	1.01 (0.94-1.08)	0.993
	06:00	0.99 (0.95-1.03)	1.000	0.93 (0.88-0.97)	0.512	1.08 (1.01-1.15)	0.993
	07:00	1.19 (1.15-1.23)	<0.001	1.1 (1.05-1.15)	0.010	1.17 (1.1-1.25)	<0.001
	08:00	1.68 (1.63-1.74)	<0.001	1.49 (1.43-1.55)	<0.001	1.36 (1.28-1.44)	<0.001
	09:00	1.29 (1.25-1.34)	<0.001	1.13 (1.08-1.18)	<0.001	1.28 (1.21-1.36)	<0.001
	10:00	1.38 (1.33-1.43)	<0.001	1.19 (1.14-1.24)	<0.001	1.31 (1.24-1.39)	<0.001
	11:00	1.14 (1.1-1.19)	<0.001	1 (0.96-1.05)	0.998	1.27 (1.2-1.35)	<0.001
	12:00	1.35 (1.3-1.4)	<0.001	1.22 (1.17-1.28)	<0.001	1.26 (1.19-1.34)	<0.001
	13:00	1.03 (1-1.07)	1.000	0.94 (0.9-0.99)	0.998	1.17 (1.1-1.25)	0.001
	14:00	1.06 (1.02-1.1)	0.515	0.97 (0.93-1.02)	0.998	1.16 (1.09-1.23)	0.004
	15:00	1.11 (1.07-1.15)	<0.001	1.02 (0.98-1.07)	0.998	1.19 (1.12-1.27)	<0.001
	16:00	1.01 (0.98-1.05)	1.000	0.96 (0.91-1)	0.998	1.15 (1.08-1.23)	0.010
	17:00	0.92 (0.88-0.96)	0.015	0.89 (0.84-0.93)	0.001	1.06 (0.99-1.13)	0.993
	18:00	0.95 (0.91-0.99)	1.000	0.92 (0.87-0.96)	0.193	1.08 (1.01-1.15)	0.993
	19:00	0.9 (0.86-0.94)	<0.001	0.87 (0.83-0.92)	<0.001	1.04 (0.97-1.11)	0.993
	20:00	1.03 (0.99-1.07)	1.000	1.01 (0.97-1.06)	0.998	1.05 (0.98-1.12)	0.993
	21:00	0.91 (0.88-0.95)	0.002	0.89 (0.85-0.94)	0.002	1.01 (0.94-1.08)	0.993
	22:00	0.96 (0.92-1)	1.000	0.93 (0.89-0.98)	0.969	1.03 (0.96-1.1)	0.993
	23:00	0.96 (0.92-1)	1.000	0.94 (0.89-0.98)	0.998	1.04 (0.97-1.11)	0.993
CAD		1		1		1	
	01:00	0.91 (0.87-0.96)	0.036	0.92 (0.88-0.97)	0.345	1.06 (0.95-1.18)	0.993
	02:00	0.93 (0.89-0.98)	0.780	0.94 (0.89-0.98)	0.998	1.1 (0.99-1.22)	0.993
	03:00	0.91 (0.87-0.95)	0.027	0.91 (0.87-0.96)	0.074	1.05 (0.94-1.17)	0.993
	04:00	0.86 (0.82-0.9)	<0.001	0.88 (0.84-0.92)	<0.001	1.02 (0.92-1.13)	0.993
	05:00	0.81 (0.77-0.85)	<0.001	0.83 (0.79-0.88)	<0.001	1.01 (0.91-1.13)	0.993
	06:00	0.91 (0.87-0.95)	0.025	0.9 (0.86-0.94)	0.008	1.08 (0.98-1.2)	0.993
	07:00	1.01 (0.97-1.05)	1.000	0.95 (0.91-1)	0.998	1.13 (1.03-1.25)	0.993
	08:00	1.38 (1.33-1.44)	<0.001	1.25 (1.2-1.3)	<0.001	1.2 (1.1-1.32)	0.059
	09:00	0.94 (0.9-0.98)	0.754	0.87 (0.83-0.91)	<0.001	1.08 (0.98-1.19)	0.993
	10:00	0.97 (0.93-1.01)	1.000	0.87 (0.83-0.91)	<0.001	1.11 (1.01-1.22)	0.993
	11:00	0.74 (0.7-0.77)	<0.001	0.71 (0.67-0.74)	<0.001	0.98 (0.89-1.09)	0.993
	12:00	0.93 (0.89-0.97)	0.335	0.87 (0.83-0.91)	<0.001	1 (0.9-1.1)	0.993
	13:00	0.68 (0.65-0.71)	<0.001	0.68 (0.65-0.72)	<0.001	0.86 (0.78-0.96)	0.993

	14:00	0.73 (0.7-0.76)	<0.001	0.71 (0.67-0.75)	<0.001	0.92 (0.83-1.02)	0.993
	15:00	0.77 (0.74-0.81)	<0.001	0.77 (0.73-0.81)	<0.001	0.85 (0.77-0.95)	0.993
	16:00	0.73 (0.7-0.77)	<0.001	0.73 (0.69-0.77)	<0.001	0.96 (0.87-1.07)	0.993
	17:00	0.73 (0.7-0.77)	<0.001	0.75 (0.71-0.79)	<0.001	0.94 (0.85-1.05)	0.993
	18:00	0.82 (0.78-0.86)	<0.001	0.83 (0.79-0.87)	<0.001	0.99 (0.89-1.1)	0.993
	19:00	0.79 (0.75-0.82)	<0.001	0.79 (0.75-0.83)	<0.001	1.02 (0.92-1.14)	0.993
	20:00	0.94 (0.9-0.98)	1.000	0.93 (0.89-0.98)	0.998	1.02 (0.92-1.13)	0.993
	21:00	0.91 (0.87-0.95)	0.018	0.91 (0.87-0.96)	0.068	1.08 (0.97-1.2)	0.993
	22:00	0.96 (0.92-1)	1.000	0.95 (0.9-1)	0.998	1.09 (0.98-1.21)	0.993
	23:00	0.98 (0.94-1.02)	1.000	0.98 (0.93-1.03)	0.998	1.11 (1-1.24)	0.993
No CAD		1		1		1	
	01:00	0.87 (0.84-0.9)	<0.001	0.85 (0.81-0.89)	<0.001	0.96 (0.9-1.02)	0.993
	02:00	0.9 (0.87-0.94)	<0.001	0.87 (0.84-0.92)	<0.001	0.99 (0.93-1.05)	0.993
	03:00	0.91 (0.88-0.94)	<0.001	0.87 (0.83-0.91)	<0.001	1.01 (0.95-1.07)	0.993
	04:00	0.86 (0.83-0.9)	<0.001	0.83 (0.79-0.87)	<0.001	0.99 (0.93-1.05)	0.993
	05:00	0.84 (0.81-0.87)	<0.001	0.79 (0.75-0.83)	<0.001	0.98 (0.93-1.04)	0.993
	06:00	0.97 (0.94-1)	1.000	0.91 (0.86-0.95)	0.008	1.03 (0.97-1.09)	0.993
	07:00	1.15 (1.11-1.19)	<0.001	1.07 (1.02-1.11)	0.998	1.1 (1.04-1.16)	0.535
	08:00	1.72 (1.67-1.77)	<0.001	1.57 (1.51-1.63)	<0.001	1.3 (1.24-1.37)	<0.001
	09:00	1.32 (1.28-1.37)	<0.001	1.19 (1.14-1.24)	<0.001	1.22 (1.16-1.28)	<0.001
	10:00	1.42 (1.38-1.47)	<0.001	1.25 (1.2-1.31)	<0.001	1.26 (1.2-1.33)	<0.001
	11:00	1.17 (1.13-1.21)	<0.001	1.05 (1-1.1)	0.998	1.2 (1.14-1.26)	<0.001
	12:00	1.42 (1.38-1.47)	<0.001	1.32 (1.27-1.38)	<0.001	1.24 (1.17-1.3)	<0.001
	13:00	1.07 (1.03-1.1)	0.123	0.99 (0.94-1.03)	0.998	1.12 (1.06-1.19)	0.019
	14:00	1.11 (1.08-1.15)	<0.001	1.04 (1-1.09)	0.998	1.13 (1.07-1.19)	0.013
	15:00	1.15 (1.11-1.19)	<0.001	1.08 (1.03-1.13)	0.234	1.15 (1.09-1.22)	<0.001
	16:00	1.02 (0.98-1.05)	1.000	0.99 (0.94-1.03)	0.998	1.07 (1.01-1.13)	0.993
	17:00	0.92 (0.89-0.96)	0.009	0.89 (0.85-0.94)	0.001	1.02 (0.96-1.08)	0.993
	18:00	0.94 (0.9-0.97)	0.127	0.9 (0.86-0.94)	0.005	1.03 (0.97-1.09)	0.993
	19:00	0.9 (0.87-0.94)	<0.001	0.89 (0.85-0.93)	<0.001	0.99 (0.94-1.05)	0.993
	20:00	1 (0.97-1.04)	1.000	0.99 (0.94-1.03)	0.998	1.01 (0.96-1.07)	0.993
	21:00	0.88 (0.84-0.91)	<0.001	0.85 (0.81-0.89)	<0.001	0.96 (0.91-1.02)	0.993
	22:00	0.94 (0.9-0.97)	0.138	0.91 (0.87-0.95)	0.022	0.98 (0.93-1.04)	0.993
	23:00	0.9 (0.87-0.94)	<0.001	0.87 (0.83-0.91)	<0.001	0.98 (0.93-1.04)	0.993
Beta-blockers		1		1		1	
	01:00	0.89 (0.85-0.92)	<0.001	0.89 (0.85-0.93)	0.001	1 (0.91-1.09)	0.993
	02:00	0.9 (0.86-0.93)	<0.001	0.9 (0.86-0.95)	0.005	0.99 (0.91-1.08)	0.993
	03:00	0.89 (0.85-0.93)	<0.001	0.88 (0.84-0.92)	<0.001	1.01 (0.93-1.11)	0.993
	04:00	0.83 (0.8-0.87)	<0.001	0.85 (0.81-0.89)	<0.001	1 (0.91-1.09)	0.993
	05:00	0.79 (0.76-0.83)	<0.001	0.8 (0.76-0.84)	<0.001	1 (0.92-1.09)	0.993
	06:00	0.88 (0.84-0.91)	<0.001	0.86 (0.82-0.9)	<0.001	1.01 (0.93-1.1)	0.993
	07:00	0.97 (0.93-1.01)	1.000	0.92 (0.88-0.96)	0.060	1.02 (0.94-1.11)	0.993
	08:00	1.38 (1.33-1.43)	<0.001	1.26 (1.21-1.31)	<0.001	1.13 (1.05-1.22)	0.993
	09:00	0.92 (0.88-0.96)	0.009	0.85 (0.81-0.88)	<0.001	0.99 (0.92-1.08)	0.993
	10:00	0.95 (0.91-0.98)	1.000	0.85 (0.81-0.88)	<0.001	1.02 (0.95-1.11)	0.993
	11:00	0.72 (0.69-0.75)	<0.001	0.68 (0.65-0.72)	<0.001	0.88 (0.81-0.96)	0.993
	12:00	0.95 (0.91-0.99)	1.000	0.89 (0.85-0.93)	<0.001	0.95 (0.88-1.03)	0.993
	13:00	0.68 (0.65-0.71)	<0.001	0.66 (0.63-0.7)	<0.001	0.86 (0.79-0.94)	0.376
	14:00	0.74 (0.71-0.77)	<0.001	0.72 (0.69-0.76)	<0.001	0.86 (0.79-0.94)	0.625

	15:00	0.79 (0.76-0.82)	<0.001	0.77 (0.73-0.8)	<0.001	0.88 (0.81-0.96)	0.993
	16:00	0.73 (0.7-0.76)	<0.001	0.72 (0.68-0.75)	<0.001	0.92 (0.84-1)	0.993
	17:00	0.74 (0.71-0.77)	<0.001	0.74 (0.7-0.77)	<0.001	0.94 (0.86-1.02)	0.993
	18:00	0.8 (0.76-0.83)	<0.001	0.8 (0.76-0.84)	<0.001	0.95 (0.87-1.03)	0.993
	19:00	0.77 (0.74-0.8)	<0.001	0.76 (0.73-0.8)	<0.001	0.98 (0.9-1.07)	0.993
	20:00	0.92 (0.88-0.95)	0.009	0.92 (0.88-0.96)	0.044	0.96 (0.88-1.04)	0.993
	21:00	0.87 (0.84-0.91)	<0.001	0.88 (0.84-0.92)	<0.001	0.97 (0.89-1.06)	0.993
	22:00	0.94 (0.9-0.98)	0.780	0.93 (0.89-0.97)	0.681	1.02 (0.94-1.11)	0.993
	23:00	0.96 (0.92-1)	1.000	0.96 (0.92-1.01)	0.998	1.05 (0.96-1.14)	0.993
No Beta-blockers		1		1		1	
	01:00	0.89 (0.85-0.92)	<0.001	0.87 (0.83-0.91)	<0.001	0.98 (0.91-1.04)	0.993
	02:00	0.93 (0.9-0.97)	0.169	0.9 (0.86-0.95)	0.014	1.02 (0.96-1.09)	0.993
	03:00	0.93 (0.89-0.97)	0.101	0.89 (0.85-0.94)	0.004	1.02 (0.95-1.08)	0.993
	04:00	0.88 (0.85-0.92)	<0.001	0.86 (0.82-0.91)	<0.001	0.99 (0.93-1.06)	0.993
	05:00	0.86 (0.83-0.9)	<0.001	0.83 (0.78-0.87)	<0.001	0.98 (0.92-1.05)	0.993
	06:00	1.01 (0.97-1.05)	1.000	0.96 (0.91-1)	0.998	1.05 (0.99-1.12)	0.993
	07:00	1.21 (1.17-1.26)	<0.001	1.13 (1.08-1.18)	<0.001	1.15 (1.09-1.22)	0.002
	08:00	1.78 (1.72-1.83)	<0.001	1.61 (1.55-1.68)	<0.001	1.35 (1.28-1.43)	<0.001
	09:00	1.41 (1.36-1.46)	<0.001	1.27 (1.21-1.33)	<0.001	1.28 (1.21-1.36)	<0.001
	10:00	1.51 (1.46-1.57)	<0.001	1.35 (1.29-1.41)	<0.001	1.33 (1.26-1.41)	<0.001
	11:00	1.26 (1.21-1.3)	<0.001	1.13 (1.08-1.19)	<0.001	1.29 (1.22-1.36)	<0.001
	12:00	1.49 (1.44-1.54)	<0.001	1.38 (1.32-1.44)	<0.001	1.3 (1.23-1.37)	<0.001
	13:00	1.13 (1.09-1.17)	<0.001	1.06 (1.01-1.11)	0.998	1.17 (1.1-1.24)	<0.001
	14:00	1.17 (1.12-1.21)	<0.001	1.08 (1.03-1.13)	0.408	1.19 (1.12-1.26)	<0.001
	15:00	1.2 (1.16-1.24)	<0.001	1.14 (1.08-1.19)	<0.001	1.19 (1.12-1.26)	<0.001
	16:00	1.07 (1.03-1.11)	0.287	1.04 (0.99-1.09)	0.998	1.11 (1.05-1.18)	0.351
	17:00	0.95 (0.92-0.99)	1.000	0.93 (0.89-0.98)	0.998	1.03 (0.97-1.1)	0.993
	18:00	0.98 (0.94-1.01)	1.000	0.95 (0.9-1)	0.998	1.06 (1-1.13)	0.993
	19:00	0.94 (0.9-0.98)	0.562	0.94 (0.89-0.98)	0.998	1.01 (0.95-1.08)	0.993
	20:00	1.03 (0.99-1.07)	1.000	1.01 (0.97-1.06)	0.998	1.04 (0.98-1.11)	0.993
	21:00	0.91 (0.87-0.94)	<0.001	0.88 (0.83-0.92)	<0.001	1 (0.94-1.07)	0.993
	22:00	0.95 (0.92-0.99)	1.000	0.92 (0.88-0.97)	0.470	1 (0.94-1.07)	0.993
	23:00	0.91 (0.87-0.94)	0.001	0.87 (0.83-0.91)	<0.001	0.99 (0.93-1.06)	0.993
Ca-inhibitor		1		1		1	
	01:00	0.92 (0.86-0.98)	1.000	0.96 (0.9-1.04)	0.998	0.88 (0.77-1)	0.993
	02:00	0.92 (0.87-0.98)	1.000	0.93 (0.87-1)	0.998	0.99 (0.87-1.12)	0.993
	03:00	0.92 (0.86-0.98)	1.000	0.94 (0.87-1.01)	0.998	0.93 (0.81-1.06)	0.993
	04:00	0.85 (0.79-0.9)	<0.001	0.87 (0.81-0.94)	0.116	0.93 (0.82-1.06)	0.993
	05:00	0.82 (0.77-0.87)	<0.001	0.82 (0.76-0.89)	<0.001	0.99 (0.87-1.13)	0.993
	06:00	0.92 (0.86-0.98)	1.000	0.91 (0.85-0.98)	0.998	0.98 (0.86-1.11)	0.993
	07:00	1 (0.94-1.06)	1.000	0.97 (0.9-1.03)	0.998	0.97 (0.86-1.1)	0.993
	08:00	1.43 (1.36-1.51)	<0.001	1.3 (1.22-1.39)	<0.001	1.15 (1.03-1.29)	0.993
	09:00	1.03 (0.97-1.09)	1.000	0.94 (0.87-1.01)	0.998	1.08 (0.96-1.21)	0.993
	10:00	1.05 (0.99-1.11)	1.000	0.92 (0.86-0.99)	0.998	1.11 (0.99-1.25)	0.993
	11:00	0.82 (0.77-0.87)	<0.001	0.75 (0.69-0.81)	<0.001	1.03 (0.91-1.16)	0.993
	12:00	1.02 (0.96-1.08)	1.000	0.93 (0.86-0.99)	0.998	1.06 (0.94-1.19)	0.993
	13:00	0.76 (0.71-0.81)	<0.001	0.74 (0.68-0.8)	<0.001	0.91 (0.8-1.03)	0.993
	14:00	0.79 (0.74-0.84)	<0.001	0.74 (0.69-0.8)	<0.001	0.93 (0.82-1.05)	0.993
	15:00	0.81 (0.76-0.86)	<0.001	0.79 (0.74-0.85)	<0.001	0.85 (0.75-0.97)	0.993

	16:00	0.73 (0.69-0.78)	<0.001	0.73 (0.67-0.79)	<0.001	0.86 (0.75-0.97)	0.993
	17:00	0.72 (0.68-0.78)	<0.001	0.74 (0.68-0.8)	<0.001	0.84 (0.73-0.96)	0.993
	18:00	0.79 (0.74-0.85)	<0.001	0.82 (0.76-0.88)	<0.001	0.84 (0.74-0.96)	0.993
	19:00	0.79 (0.74-0.84)	<0.001	0.8 (0.74-0.87)	<0.001	0.91 (0.79-1.03)	0.993
	20:00	0.93 (0.87-0.98)	1.000	0.93 (0.86-1)	0.998	0.94 (0.83-1.07)	0.993
	21:00	0.88 (0.82-0.93)	0.017	0.9 (0.83-0.96)	0.915	0.91 (0.79-1.03)	0.993
	22:00	0.95 (0.89-1.01)	1.000	0.96 (0.89-1.03)	0.998	0.94 (0.83-1.07)	0.993
	23:00	0.95 (0.89-1.01)	1.000	0.96 (0.89-1.03)	0.998	0.98 (0.87-1.12)	0.993
No Ca-inhibitor		1		1		1	
	01:00	0.88 (0.85-0.91)	<0.001	0.86 (0.83-0.89)	<0.001	1 (0.95-1.06)	0.993
	02:00	0.91 (0.89-0.94)	<0.001	0.89 (0.86-0.93)	<0.001	1.02 (0.96-1.08)	0.993
	03:00	0.91 (0.88-0.94)	<0.001	0.87 (0.84-0.91)	<0.001	1.03 (0.98-1.09)	0.993
	04:00	0.86 (0.84-0.89)	<0.001	0.85 (0.82-0.88)	<0.001	1.01 (0.95-1.06)	0.993
	05:00	0.83 (0.8-0.86)	<0.001	0.81 (0.78-0.84)	<0.001	0.99 (0.93-1.05)	0.993
	06:00	0.95 (0.92-0.98)	0.770	0.9 (0.87-0.93)	<0.001	1.05 (1-1.11)	0.993
	07:00	1.12 (1.09-1.15)	<0.001	1.03 (0.99-1.06)	0.998	1.13 (1.07-1.19)	0.002
	08:00	1.62 (1.58-1.67)	<0.001	1.45 (1.41-1.5)	<0.001	1.3 (1.24-1.37)	<0.001
	09:00	1.21 (1.17-1.24)	<0.001	1.07 (1.03-1.11)	0.083	1.2 (1.15-1.27)	<0.001
	10:00	1.29 (1.25-1.32)	<0.001	1.12 (1.08-1.16)	<0.001	1.25 (1.19-1.31)	<0.001
	11:00	1.04 (1.01-1.07)	1.000	0.93 (0.9-0.97)	0.063	1.17 (1.11-1.23)	<0.001
	12:00	1.28 (1.24-1.31)	<0.001	1.17 (1.13-1.21)	<0.001	1.2 (1.14-1.26)	<0.001
	13:00	0.95 (0.92-0.98)	0.478	0.88 (0.84-0.91)	<0.001	1.09 (1.04-1.15)	0.684
	14:00	1 (0.97-1.03)	1.000	0.93 (0.89-0.96)	0.037	1.11 (1.05-1.17)	0.088
	15:00	1.05 (1.02-1.08)	0.960	0.98 (0.94-1.01)	0.998	1.13 (1.07-1.19)	0.005
	16:00	0.95 (0.92-0.98)	0.231	0.9 (0.87-0.94)	<0.001	1.08 (1.03-1.14)	0.993
	17:00	0.88 (0.85-0.91)	<0.001	0.85 (0.82-0.88)	<0.001	1.03 (0.98-1.09)	0.993
	18:00	0.91 (0.89-0.94)	<0.001	0.88 (0.85-0.92)	<0.001	1.05 (1-1.11)	0.993
	19:00	0.87 (0.85-0.9)	<0.001	0.85 (0.82-0.89)	<0.001	1.02 (0.96-1.08)	0.993
	20:00	0.99 (0.96-1.02)	1.000	0.97 (0.93-1.01)	0.998	1.03 (0.97-1.09)	0.993
	21:00	0.89 (0.87-0.92)	<0.001	0.87 (0.84-0.91)	<0.001	1.01 (0.95-1.06)	0.993
	22:00	0.95 (0.92-0.98)	0.174	0.92 (0.89-0.96)	0.005	1.02 (0.97-1.08)	0.993
	23:00	0.93 (0.9-0.96)	0.003	0.91 (0.87-0.94)	<0.001	1.02 (0.96-1.08)	0.993
Aspirin		1		1		1	
	01:00	0.87 (0.84-0.91)	<0.001	0.88 (0.84-0.92)	<0.001	0.97 (0.88-1.06)	0.993
	02:00	0.89 (0.86-0.93)	<0.001	0.9 (0.86-0.94)	0.001	1 (0.91-1.09)	0.993
	03:00	0.88 (0.85-0.92)	<0.001	0.88 (0.84-0.92)	<0.001	0.99 (0.91-1.09)	0.993
	04:00	0.81 (0.78-0.84)	<0.001	0.82 (0.79-0.86)	<0.001	0.96 (0.88-1.05)	0.993
	05:00	0.77 (0.74-0.8)	<0.001	0.78 (0.75-0.82)	<0.001	0.96 (0.87-1.05)	0.993
	06:00	0.88 (0.85-0.92)	<0.001	0.87 (0.83-0.91)	<0.001	1.02 (0.93-1.11)	0.993
	07:00	1.01 (0.97-1.05)	1.000	0.95 (0.91-0.99)	0.998	1.09 (1.01-1.19)	0.993
	08:00	1.42 (1.37-1.47)	<0.001	1.28 (1.23-1.33)	<0.001	1.22 (1.13-1.31)	<0.001
	09:00	0.96 (0.92-0.99)	1.000	0.89 (0.85-0.93)	<0.001	1.03 (0.95-1.11)	0.993
	10:00	0.98 (0.95-1.02)	1.000	0.89 (0.85-0.93)	<0.001	1.05 (0.97-1.14)	0.993
	11:00	0.75 (0.72-0.78)	<0.001	0.71 (0.68-0.75)	<0.001	0.93 (0.85-1.01)	0.993
	12:00	0.97 (0.93-1.01)	1.000	0.91 (0.87-0.95)	0.004	0.98 (0.9-1.07)	0.993
	13:00	0.69 (0.67-0.73)	<0.001	0.69 (0.66-0.73)	<0.001	0.83 (0.76-0.91)	0.038
	14:00	0.75 (0.72-0.78)	<0.001	0.74 (0.7-0.77)	<0.001	0.87 (0.8-0.95)	0.993
	15:00	0.78 (0.75-0.81)	<0.001	0.77 (0.74-0.81)	<0.001	0.83 (0.76-0.91)	0.037
	16:00	0.73 (0.7-0.76)	<0.001	0.73 (0.7-0.77)	<0.001	0.87 (0.79-0.95)	0.993

	17:00	0.71 (0.68-0.74)	<0.001	0.72 (0.68-0.76)	<0.001	0.89 (0.81-0.97)	0.993
	18:00	0.8 (0.76-0.83)	<0.001	0.81 (0.77-0.85)	<0.001	0.91 (0.83-1)	0.993
	19:00	0.76 (0.73-0.8)	<0.001	0.76 (0.73-0.8)	<0.001	0.95 (0.87-1.04)	0.993
	20:00	0.92 (0.88-0.96)	0.014	0.92 (0.88-0.96)	0.049	0.97 (0.89-1.06)	0.993
	21:00	0.86 (0.83-0.9)	<0.001	0.87 (0.83-0.91)	<0.001	0.96 (0.88-1.05)	0.993
	22:00	0.93 (0.89-0.97)	0.095	0.92 (0.88-0.96)	0.108	1.01 (0.93-1.1)	0.993
	23:00	0.94 (0.9-0.97)	0.363	0.94 (0.9-0.98)	0.998	1 (0.92-1.1)	0.993
No Aspirin		1		1		1	
	01:00	0.9 (0.86-0.94)	<0.001	0.88 (0.83-0.92)	<0.001	0.99 (0.93-1.06)	0.993
	02:00	0.94 (0.9-0.98)	0.480	0.91 (0.87-0.96)	0.067	1.02 (0.96-1.09)	0.993
	03:00	0.94 (0.9-0.97)	0.416	0.9 (0.85-0.94)	0.010	1.03 (0.96-1.09)	0.993
	04:00	0.91 (0.87-0.95)	0.001	0.89 (0.85-0.94)	0.004	1.01 (0.95-1.08)	0.993
	05:00	0.88 (0.85-0.92)	<0.001	0.84 (0.8-0.89)	<0.001	1.01 (0.95-1.07)	0.993
	06:00	1.01 (0.97-1.05)	1.000	0.95 (0.9-0.99)	0.998	1.05 (0.99-1.12)	0.993
	07:00	1.18 (1.14-1.22)	<0.001	1.1 (1.05-1.15)	0.040	1.11 (1.05-1.18)	0.234
	08:00	1.74 (1.69-1.8)	<0.001	1.59 (1.53-1.66)	<0.001	1.31 (1.24-1.38)	<0.001
	09:00	1.37 (1.33-1.42)	<0.001	1.23 (1.17-1.28)	<0.001	1.26 (1.2-1.33)	<0.001
	10:00	1.49 (1.44-1.54)	<0.001	1.31 (1.25-1.37)	<0.001	1.32 (1.25-1.39)	<0.001
	11:00	1.24 (1.19-1.28)	<0.001	1.11 (1.06-1.16)	0.010	1.26 (1.19-1.33)	<0.001
	12:00	1.47 (1.42-1.53)	<0.001	1.37 (1.31-1.43)	<0.001	1.28 (1.21-1.35)	<0.001
	13:00	1.12 (1.08-1.17)	<0.001	1.03 (0.98-1.09)	0.998	1.18 (1.11-1.25)	<0.001
	14:00	1.16 (1.12-1.21)	<0.001	1.07 (1.02-1.13)	0.998	1.18 (1.12-1.25)	<0.001
	15:00	1.21 (1.17-1.26)	<0.001	1.14 (1.09-1.2)	<0.001	1.21 (1.14-1.28)	<0.001
	16:00	1.07 (1.03-1.11)	0.141	1.03 (0.98-1.08)	0.998	1.14 (1.07-1.21)	0.020
	17:00	0.98 (0.94-1.02)	1.000	0.96 (0.91-1.01)	0.998	1.06 (0.99-1.12)	0.993
	18:00	0.98 (0.94-1.02)	1.000	0.94 (0.89-0.99)	0.998	1.07 (1.01-1.14)	0.993
	19:00	0.95 (0.91-0.99)	1.000	0.94 (0.89-0.98)	0.998	1.03 (0.96-1.09)	0.993
	20:00	1.03 (0.99-1.07)	1.000	1.01 (0.97-1.06)	0.998	1.04 (0.98-1.1)	0.993
	21:00	0.92 (0.88-0.95)	0.006	0.89 (0.85-0.94)	0.002	1 (0.94-1.07)	0.993
	22:00	0.96 (0.93-1)	1.000	0.94 (0.89-0.98)	0.998	1.01 (0.95-1.07)	0.993
	23:00	0.93 (0.9-0.97)	0.156	0.89 (0.85-0.93)	0.002	1.02 (0.96-1.08)	0.993
ACE-I/ARB		1		1		1	
	01:00	0.87 (0.83-0.91)	<0.001	0.9 (0.85-0.95)	0.026	0.9 (0.81-1.01)	0.993
	02:00	0.87 (0.83-0.92)	<0.001	0.89 (0.84-0.94)	0.009	0.93 (0.84-1.03)	0.993
	03:00	0.87 (0.83-0.91)	<0.001	0.89 (0.84-0.94)	0.011	0.89 (0.8-0.99)	0.993
	04:00	0.81 (0.77-0.85)	<0.001	0.84 (0.79-0.88)	<0.001	0.92 (0.83-1.02)	0.993
	05:00	0.78 (0.75-0.82)	<0.001	0.82 (0.77-0.86)	<0.001	0.9 (0.81-1)	0.993
	06:00	0.84 (0.8-0.88)	<0.001	0.83 (0.79-0.88)	<0.001	0.91 (0.83-1.01)	0.993
	07:00	1.03 (0.98-1.08)	1.000	0.99 (0.94-1.05)	0.998	1.02 (0.93-1.13)	0.993
	08:00	1.48 (1.42-1.55)	<0.001	1.37 (1.31-1.43)	<0.001	1.16 (1.06-1.27)	0.839
	09:00	1.01 (0.96-1.05)	1.000	0.94 (0.89-0.98)	0.998	1.07 (0.97-1.17)	0.993
	10:00	1.03 (0.98-1.08)	1.000	0.94 (0.89-0.99)	0.998	1.06 (0.97-1.16)	0.993
	11:00	0.79 (0.75-0.83)	<0.001	0.75 (0.71-0.8)	<0.001	0.97 (0.88-1.07)	0.993
	12:00	1.04 (0.99-1.08)	1.000	0.97 (0.92-1.02)	0.998	1.03 (0.94-1.13)	0.993
	13:00	0.74 (0.7-0.78)	<0.001	0.73 (0.69-0.77)	<0.001	0.9 (0.81-0.99)	0.993
	14:00	0.78 (0.74-0.82)	<0.001	0.76 (0.72-0.81)	<0.001	0.92 (0.83-1.01)	0.993
	15:00	0.82 (0.78-0.86)	<0.001	0.81 (0.76-0.85)	<0.001	0.9 (0.81-0.99)	0.993
	16:00	0.74 (0.7-0.78)	<0.001	0.75 (0.7-0.79)	<0.001	0.85 (0.77-0.94)	0.993
	17:00	0.72 (0.68-0.76)	<0.001	0.73 (0.69-0.77)	<0.001	0.9 (0.81-0.99)	0.993

	18:00	0.78 (0.74-0.82)	<0.001	0.8 (0.75-0.84)	<0.001	0.87 (0.78-0.97)	0.993
	19:00	0.77 (0.74-0.81)	<0.001	0.79 (0.74-0.83)	<0.001	0.91 (0.82-1.01)	0.993
	20:00	0.93 (0.88-0.97)	0.484	0.93 (0.89-0.98)	0.998	0.94 (0.84-1.04)	0.993
	21:00	0.83 (0.79-0.87)	<0.001	0.83 (0.78-0.87)	<0.001	0.94 (0.85-1.05)	0.993
	22:00	0.91 (0.86-0.95)	0.016	0.93 (0.88-0.98)	0.998	0.87 (0.78-0.97)	0.993
	23:00	0.92 (0.88-0.96)	0.141	0.94 (0.89-0.99)	0.998	0.93 (0.84-1.04)	0.993
No ACE-I/ARB		1		1		1	
	01:00	0.89 (0.86-0.93)	<0.001	0.87 (0.83-0.91)	<0.001	1.01 (0.95-1.07)	0.993
	02:00	0.94 (0.91-0.97)	0.111	0.91 (0.87-0.95)	0.007	1.04 (0.98-1.1)	0.993
	03:00	0.93 (0.9-0.96)	0.021	0.88 (0.85-0.92)	<0.001	1.06 (1-1.12)	0.993
	04:00	0.89 (0.86-0.92)	<0.001	0.87 (0.83-0.9)	<0.001	1.02 (0.96-1.08)	0.993
	05:00	0.85 (0.82-0.88)	<0.001	0.81 (0.77-0.85)	<0.001	1.02 (0.96-1.08)	0.993
	06:00	1 (0.97-1.04)	1.000	0.95 (0.91-0.99)	0.998	1.08 (1.02-1.14)	0.993
	07:00	1.13 (1.09-1.17)	<0.001	1.03 (0.99-1.07)	0.998	1.13 (1.07-1.2)	0.005
	08:00	1.64 (1.59-1.69)	<0.001	1.45 (1.4-1.51)	<0.001	1.32 (1.25-1.38)	<0.001
	09:00	1.26 (1.22-1.3)	<0.001	1.11 (1.07-1.15)	<0.001	1.22 (1.16-1.29)	<0.001
	10:00	1.35 (1.31-1.4)	<0.001	1.17 (1.12-1.21)	<0.001	1.28 (1.22-1.35)	<0.001
	11:00	1.11 (1.07-1.15)	<0.001	0.98 (0.94-1.02)	0.998	1.21 (1.14-1.27)	<0.001
	12:00	1.33 (1.29-1.37)	<0.001	1.21 (1.16-1.25)	<0.001	1.23 (1.17-1.3)	<0.001
	13:00	1.01 (0.97-1.04)	1.000	0.92 (0.88-0.96)	0.102	1.12 (1.06-1.18)	0.050
	14:00	1.06 (1.02-1.09)	0.342	0.97 (0.93-1.01)	0.998	1.13 (1.07-1.2)	0.006
	15:00	1.1 (1.06-1.13)	<0.001	1.02 (0.98-1.06)	0.998	1.14 (1.08-1.21)	0.001
	16:00	1 (0.96-1.03)	1.000	0.94 (0.9-0.99)	0.998	1.11 (1.05-1.18)	0.179
	17:00	0.92 (0.89-0.95)	0.001	0.89 (0.85-0.93)	<0.001	1.04 (0.98-1.1)	0.993
	18:00	0.95 (0.92-0.98)	0.989	0.92 (0.88-0.96)	0.025	1.07 (1.01-1.13)	0.993
	19:00	0.9 (0.87-0.94)	<0.001	0.88 (0.84-0.92)	<0.001	1.03 (0.97-1.09)	0.993
	20:00	1 (0.97-1.04)	1.000	0.98 (0.94-1.02)	0.998	1.04 (0.98-1.1)	0.993
	21:00	0.92 (0.89-0.96)	0.003	0.91 (0.87-0.95)	0.005	1.01 (0.95-1.07)	0.993
	22:00	0.97 (0.94-1)	1.000	0.93 (0.89-0.97)	0.211	1.05 (0.99-1.12)	0.993
	23:00	0.94 (0.91-0.98)	0.276	0.91 (0.87-0.95)	0.003	1.04 (0.98-1.1)	0.993
Statins		1		1		1	
	01:00	0.85 (0.81-0.9)	<0.001	0.86 (0.81-0.91)	<0.001	0.95 (0.84-1.06)	0.993
	02:00	0.86 (0.82-0.91)	<0.001	0.88 (0.84-0.94)	0.010	0.91 (0.81-1.02)	0.993
	03:00	0.84 (0.79-0.88)	<0.001	0.84 (0.79-0.89)	<0.001	0.94 (0.84-1.06)	0.993
	04:00	0.8 (0.75-0.84)	<0.001	0.81 (0.76-0.86)	<0.001	0.95 (0.84-1.07)	0.993
	05:00	0.74 (0.7-0.78)	<0.001	0.76 (0.72-0.81)	<0.001	0.88 (0.78-0.99)	0.993
	06:00	0.84 (0.8-0.89)	<0.001	0.84 (0.79-0.89)	<0.001	0.93 (0.83-1.05)	0.993
	07:00	0.98 (0.93-1.03)	1.000	0.93 (0.88-0.98)	0.998	1.06 (0.95-1.18)	0.993
	08:00	1.45 (1.39-1.52)	<0.001	1.33 (1.27-1.4)	<0.001	1.17 (1.05-1.29)	0.993
	09:00	1 (0.96-1.05)	1.000	0.95 (0.9-1)	0.998	1.05 (0.95-1.17)	0.993
	10:00	1.04 (0.99-1.09)	1.000	0.94 (0.89-0.99)	0.998	1.13 (1.02-1.26)	0.993
	11:00	0.81 (0.77-0.85)	<0.001	0.77 (0.73-0.82)	<0.001	1.03 (0.92-1.14)	0.993
	12:00	1 (0.95-1.05)	1.000	0.94 (0.89-0.99)	0.998	1.02 (0.92-1.13)	0.993
	13:00	0.74 (0.7-0.78)	<0.001	0.74 (0.7-0.79)	<0.001	0.9 (0.8-1)	0.993
	14:00	0.78 (0.74-0.82)	<0.001	0.74 (0.7-0.79)	<0.001	0.98 (0.88-1.09)	0.993
	15:00	0.8 (0.76-0.84)	<0.001	0.79 (0.75-0.84)	<0.001	0.85 (0.76-0.96)	0.993
	16:00	0.73 (0.69-0.77)	<0.001	0.74 (0.7-0.79)	<0.001	0.85 (0.76-0.95)	0.993
	17:00	0.73 (0.69-0.77)	<0.001	0.73 (0.68-0.77)	<0.001	0.96 (0.86-1.08)	0.993
	18:00	0.77 (0.73-0.81)	<0.001	0.78 (0.74-0.83)	<0.001	0.89 (0.79-1)	0.993

	19:00	0.77 (0.73-0.81)	<0.001	0.79 (0.74-0.83)	<0.001	0.91 (0.81-1.02)	0.993
	20:00	0.92 (0.87-0.96)	0.253	0.92 (0.87-0.98)	0.998	0.93 (0.83-1.05)	0.993
	21:00	0.84 (0.8-0.88)	<0.001	0.85 (0.8-0.9)	<0.001	0.91 (0.81-1.02)	0.993
	22:00	0.91 (0.87-0.96)	0.095	0.92 (0.87-0.97)	0.743	0.94 (0.84-1.05)	0.993
	23:00	0.94 (0.89-0.98)	1.000	0.95 (0.9-1.01)	0.998	0.97 (0.87-1.09)	0.993
No Statins		1		1		1	
	01:00	0.9 (0.87-0.93)	<0.001	0.89 (0.85-0.93)	<0.001	0.99 (0.94-1.05)	0.993
	02:00	0.94 (0.91-0.97)	0.073	0.91 (0.88-0.95)	0.005	1.04 (0.98-1.1)	0.993
	03:00	0.94 (0.91-0.97)	0.165	0.91 (0.88-0.95)	0.007	1.03 (0.98-1.09)	0.993
	04:00	0.89 (0.86-0.92)	<0.001	0.88 (0.84-0.91)	<0.001	1.01 (0.95-1.07)	0.993
	05:00	0.87 (0.84-0.9)	<0.001	0.84 (0.8-0.87)	<0.001	1.02 (0.96-1.08)	0.993
	06:00	0.99 (0.96-1.02)	1.000	0.94 (0.9-0.97)	0.458	1.07 (1.01-1.13)	0.993
	07:00	1.14 (1.11-1.18)	<0.001	1.06 (1.02-1.1)	0.998	1.12 (1.06-1.18)	0.025
	08:00	1.64 (1.6-1.69)	<0.001	1.47 (1.42-1.52)	<0.001	1.31 (1.24-1.37)	<0.001
	09:00	1.24 (1.21-1.28)	<0.001	1.09 (1.05-1.13)	0.005	1.22 (1.16-1.28)	<0.001
	10:00	1.33 (1.29-1.37)	<0.001	1.15 (1.11-1.19)	<0.001	1.25 (1.19-1.31)	<0.001
	11:00	1.08 (1.05-1.12)	0.001	0.96 (0.92-1)	0.998	1.18 (1.12-1.24)	<0.001
	12:00	1.33 (1.29-1.37)	<0.001	1.21 (1.16-1.25)	<0.001	1.22 (1.16-1.28)	<0.001
	13:00	0.99 (0.96-1.02)	1.000	0.9 (0.86-0.94)	0.001	1.1 (1.05-1.17)	0.188
	14:00	1.04 (1.01-1.08)	1.000	0.97 (0.93-1.01)	0.998	1.1 (1.05-1.16)	0.205
	15:00	1.09 (1.05-1.12)	<0.001	1.01 (0.97-1.05)	0.998	1.14 (1.08-1.2)	0.001
	16:00	0.98 (0.95-1.01)	1.000	0.93 (0.89-0.97)	0.267	1.1 (1.04-1.16)	0.761
	17:00	0.9 (0.87-0.93)	<0.001	0.88 (0.84-0.92)	<0.001	1.01 (0.96-1.07)	0.993
	18:00	0.94 (0.91-0.97)	0.174	0.91 (0.88-0.95)	0.009	1.05 (1-1.11)	0.993
	19:00	0.9 (0.87-0.93)	<0.001	0.87 (0.84-0.91)	<0.001	1.02 (0.97-1.08)	0.993
	20:00	1 (0.97-1.04)	1.000	0.98 (0.94-1.02)	0.998	1.03 (0.98-1.09)	0.993
	21:00	0.91 (0.88-0.94)	<0.001	0.89 (0.85-0.93)	<0.001	1.01 (0.95-1.07)	0.993
	22:00	0.96 (0.93-0.99)	1.000	0.94 (0.9-0.97)	0.384	1.03 (0.97-1.08)	0.993
	23:00	0.93 (0.9-0.96)	0.019	0.9 (0.86-0.94)	<0.001	1.02 (0.97-1.08)	0.993

Supplementary table 2. Subgroup analyses.

All p-values are adjusted for multiple testing.

ACE-I= Angiotensin Converting Enzyme-Inhibitors: ARB= angiotensin receptor blocker; CAD=Coronary artery disease.

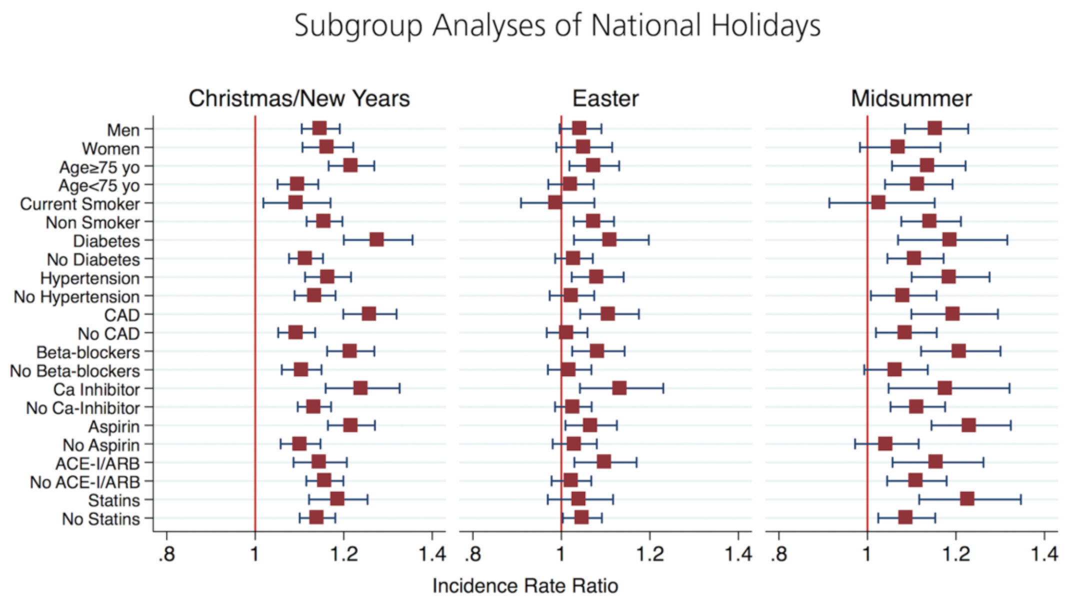

Supplementary Figure 1. Subgroup analyses.

Shown are the associated risks of overall MI in various subgroups and major national holidays expressed as incidence rate ratios. Christmas/New years include Christmas Eve, Christmas Day, Boxing Day, New Years Eve and Day as well as the epiphany. Easter holiday includes Good Friday, Easter Eve and Day and Easter Monday. P-value<0.05 for all bars not crossing the red line. P-values after adjustment for multiple testing are presented in Supplementary Table 1.

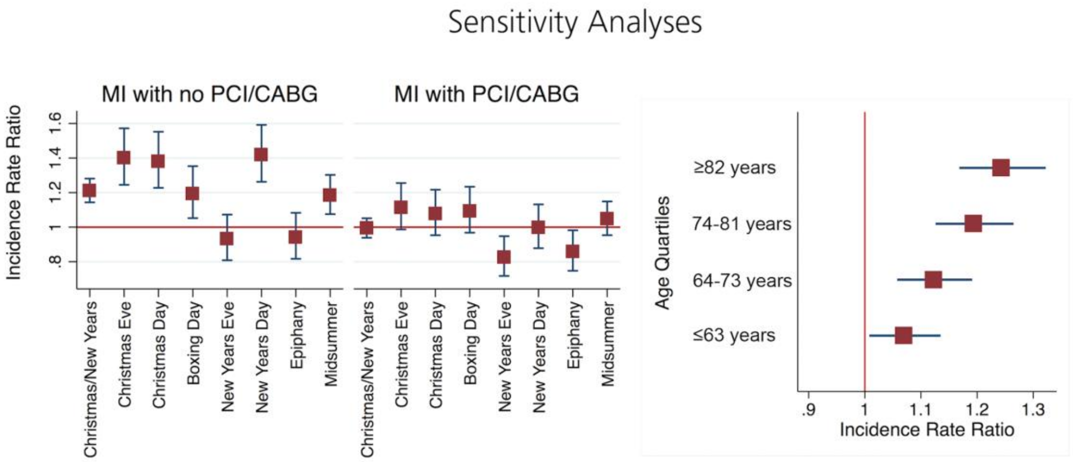

Supplementary Figure 2. Additional subgroup analyses.

Left figure shows the associated risk of MI requiring revascularization after coronary angiography was performed vs. those who did not undergo PCI/CABG on holidays. Right figure shows the associated risk of MI on Christmas/New Years' holiday for each age quartile. Christmas/New Years' include Christmas Eve, Christmas Day, Boxing Day, New Years' Eve and Day as well as the epiphany. P-value<0.05 for all bars not crossing the red line.

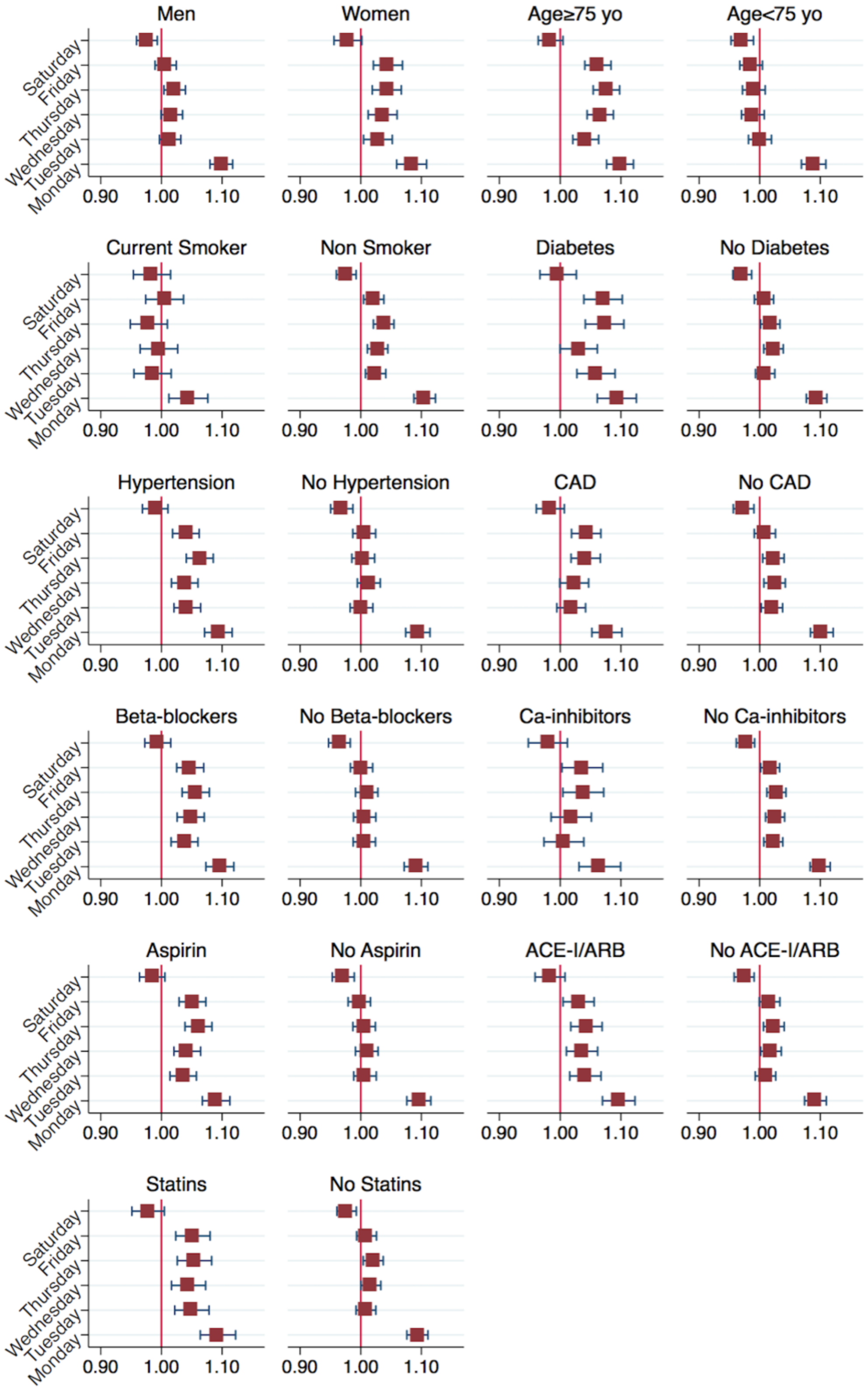

Supplementary Figure 3. Subgroup analyses of day of week.

Shown are the associated risks of overall MI in various subgroups and day of week expressed as incidence rate ratio. Reference period for day of week is Sunday and reference hour for hour of symptom onset is 00:00. P-values after adjustment for multiple testing are presented in Supplementary Table 1.

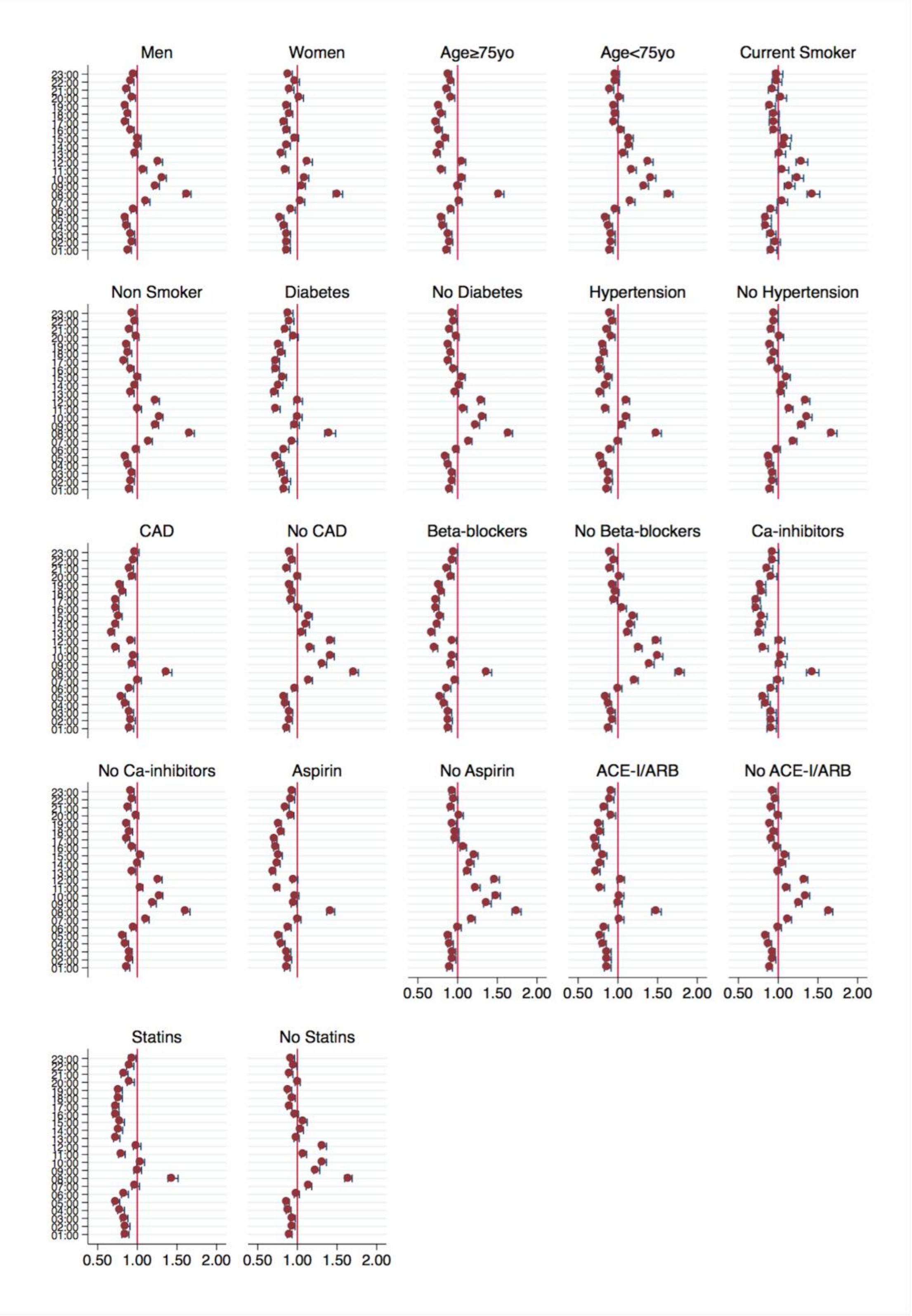

Supplementary Figure 4. Subgroup analyses of hour of symptom onset

Shown are the associated risks of overall MI in various subgroups and hour of symptom onset expressed as incidence rate ratios. Reference period is 00:00. P-values after adjustment for multiple testing are presented in Supplementary Table 1.
